# RNA binding motif protein RBM41 promotes colorectal tumorigenesis by impeding the maturation of *NDRG1* pre-mRNA

**DOI:** 10.1038/s41420-026-03197-6

**Published:** 2026-06-20

**Authors:** Yanxin Liu, Jianfeng Mu, Jiming Yu, Qirong Li, Tiantian Li, Qiang Feng, Tong Sun, Ke Sun, Yang Hao, Jinhai Yu, Dongxu Wang

**Affiliations:** 1 Department of Gastric and Colorectal Surgery, General Surgery Center, The First Hospital of Jilin University, Changchun, PR China; 2 Department of Laboratory Animals, College of Animal Sciences, Jilin University, Changchun, PR China

**Keywords:** Cancer, Molecular biology

## Abstract

Colorectal cancer (CRC) is a prevalent and highly lethal malignancy. RNA-binding motif (RBM) proteins have been demonstrated in CRC pathogenesis. However, the biological function and regulatory mechanisms of *RBM41* in CRC remain poorly understood. Here, we demonstrate that *RBM41* is significantly elevated in CRC tissues and is associated with poor prognosis in patients. Overexpression of RBM41 can enhance the malignant proliferation phenotype of CRC cells; in contrast, inhibition of *RBM41* significantly decreases CRC cell proliferation and induces autophagic cell death and apoptosis in HT29 and SW480 cells. Mechanistically, *RBM41* interferes with the processing of N-myc downregulated gene 1 (*NDRG1*) pre-mRNA by directly binding to its 3’ untranslated region (3’ UTR), thereby decreasing the mature transcript and protein levels of tumor suppressor *NDRG1*. Concurrent knockdown of *NDRG1* reversed the oncogenic functions of *RBM41* in HT29 cells and in fast-growing xenograft tumors in vivo. Moreover, patient-derived organoid (PDO) models with high *RBM41* expression exhibited increased resistance to 5-fluorouracil, oxaliplatin, and irinotecan. In summary, our findings demonstrate that *RBM41* promotes CRC progression by post-transcriptionally repressing *NDRG1*, underscoring its potential as a promising therapeutic target for CRC.

## Introduction

Colorectal cancer (CRC) is one of the leading causes of cancer-related mortality worldwide [1–3]. Despite significant advances in comprehensive treatment strategies involving surgery, chemotherapy, and targeted therapy [4], metastasis and chemoresistance remain key determinants of poor patient prognosis [5, 6]. Elucidating the molecular mechanisms underlying CRC pathogenesis is urgently needed to improve clinical outcomes.

The RNA-binding motif (RBM) protein family represents an important subclass of RNA-binding proteins (RBPs) characterized by conserved RNA-binding domains [7, 8]. RBM protein precisely regulates gene expression at the post-transcriptional level by controlling RNA splicing, stability, translation, and subcellular localization, thereby influencing critical biological processes such as cell proliferation, apoptosis, and differentiation [9–11]. Their dysregulation is increasingly being implicated in tumor initiation and progression of CRC. RBM5 has been shown to facilitate epithelial-mesenchymal transition and metastasis by regulating RNA splicing [12], while RBM39 promotes colorectal cancer metastasis by mediating the alternative splicing of CDK5RAP2 [13]. However, certain RBM proteins exert tumor-suppressive functions. RBM38 [14] and RBM24 [15] inhibit tumor progression by stabilizing the mRNA of phosphatase and tensin homolog (PTEN), whereas RBMS3 promotes the degradation of LIM Zinc Finger Domain Containing 1 (LIMS1) mRNA [16]. These findings highlight the functional diversity and significance of the RBM family in CRC. Recent studies have suggested that RBM41 is involved in minor spliceosome function [17]. We observed that *RBM41* is significantly upregulated in CRC tissues and correlates with poor patient prognosis, as determined by bioinformatic analysis of the Gene Expression Omnibus (GEO) and The Cancer Genome Atlas (TCGA) databases, suggesting that *RBM41* may act as a potential oncogene. However, the fundamental processes underlying RBM41’s regulation of CRC remain unclear.

In this study, we demonstrate that *RBM41* promotes CRC progression by overexpression and deletion in HT29, T84, and SW480 cells, and identify N-Myc Downstream Regulated 1 (NDRG1) as a critical mediator of RBM41-driven tumorigenicity. *RBM41* binds to the 3’ UTR of *NDRG1* pre-mRNA and inhibits its maturation, thereby downregulating the expression of this tumor suppressor. Elevated *RBM41* expression is associated with increased chemotherapy resistance in both cell lines and patient-derived organoid (PDO) models. Our findings reveal the oncogenic role of *RBM41* in CRC and elucidate a novel post-transcriptional regulatory mechanism.

## Results

### Upregulated *RBM41* in CRC tissues is closely associated with poor patient prognosis

**Fig. 1:**
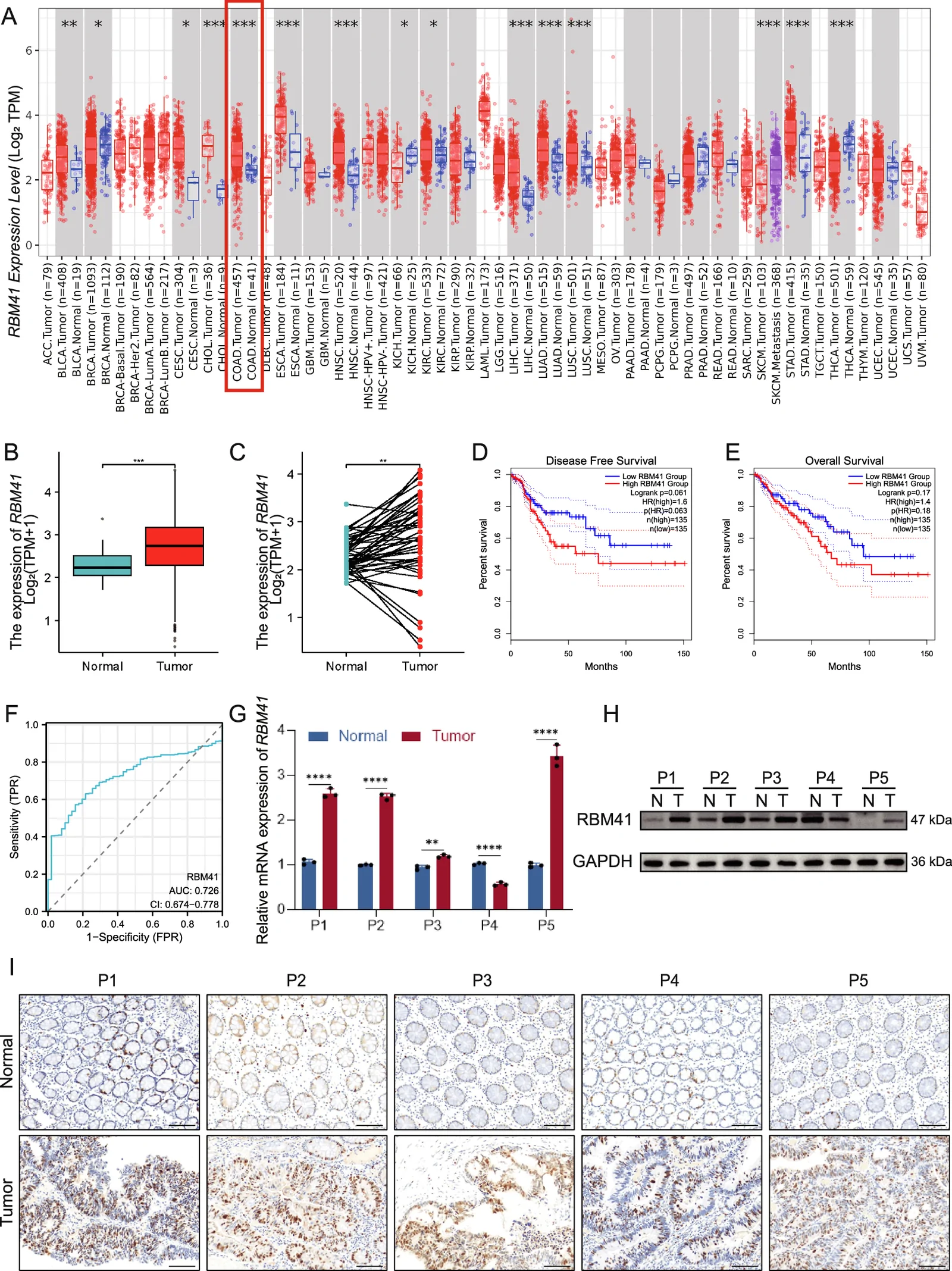
RBM41 is upregulated in colorectal cancer and correlates with poor prognosis. **A** Analysis of *RBM41* mRNA expression levels across multiple cancer types and corresponding normal tissues from the XIANTAO database. **B** Comparison of *RBM41* mRNA expression between unpaired colorectal cancer (CRC) tissues and normal tissues based on TCGA data (*n* = 619 tumor tissues, *n* = 51 normal tissues; biological replicates). **C** Comparison of *RBM41* mRNA expression between paired CRC tissues and adjacent normal tissues from the TCGA database (*n* = 50 paired samples, biological replicates). **D** Disease-free survival analysis of CRC patients stratified by high versus low *RBM41* expression (*n* = 322 high expression, *n* = 322 low expression; biological replicates). **E** Overall survival analysis of CRC patients stratified by high versus low *RBM41* expression (*n* = 322 high expression, *n* = 322 low expression; biological replicates). **F** The diagnostic value of *RBM41* for distinguishing CRC from normal tissues by the receiver operating characteristic (ROC) curve. The area under the curve (AUC) and 95% confidence interval (CI) are indicated. AUC = 0.726, 95% CI: 0.674–0.778. **G** Relative *RBM41* mRNA levels in CRC tissues and their matched adjacent normal tissues from five patients (P1–P5), as determined by RT‑qPCR. Data are presented as mean ± SD of *n* = 3 technical replicates per patient. **H** Western blot analysis of RBM41 protein levels in five pairs of CRC (T) and adjacent normal (N) tissues from five patients (P1–P5). GAPDH served as a loading control. Representative blots from *n* = 3 technical replicates per patient. **I** Immunohistochemical (IHC) staining of Ki‑67 in paired CRC and adjacent normal tissues from five representative patients. Scale bar, 100 μm. Representative images from n = 3 technical replicates per patient. Statistical significance was determined using Student’s *t*‑test (**B**, **C**, **G**) or log‑rank test (**D**, **E**). *, P < 0.05; **, P < 0.01; ***, P < 0.001; ****, P < 0.0001.

Analysis of public databases indicated that *RBM41* is aberrantly overexpressed in various cancer types (Fig. 1A), with significantly higher *RBM41* expression in CRC tissues compared to adjacent normal tissues in both unpaired and paired comparisons (Fig. 1B, 1C). We next investigated the clinical correlation between *RBM41* expression and prognosis in CRC patients. Survival analysis showed that patients with high *RBM41* expression have a shorter disease-free survival and overall survival, compared to patients with lower *RBM41* levels (Fig. 1D, E). Receiver operating characteristic (ROC) curve analysis further supported the diagnostic relevance of *RBM41* expression for CRC, with an area under the curve (AUC) value of 0.726 (Fig. 1F).

To validate these findings, we assessed *RBM41* expression in clinical samples from five CRC patients. Results showed that both mRNA and protein levels of *RBM41* were markedly upregulated in tumor tissues compared to matched adjacent tissues in four of the five patients (Fig. 1G, H). Given the association between high *RBM41* expression and poor prognosis, we sought to assess its potential functional impact on tumor cell proliferation. Immunohistochemical analysis of paired sections from five patients revealed enhanced Ki-67 staining in tumor tissues from four cases (Fig. 1I). Together, these results indicate that *RBM41* exhibits high levels in CRC and that its expression level is closely correlated with poor prognosis in patients.

### *RBM41* promotes CRC cell proliferation and inhibits cell death

**Fig. 2:**
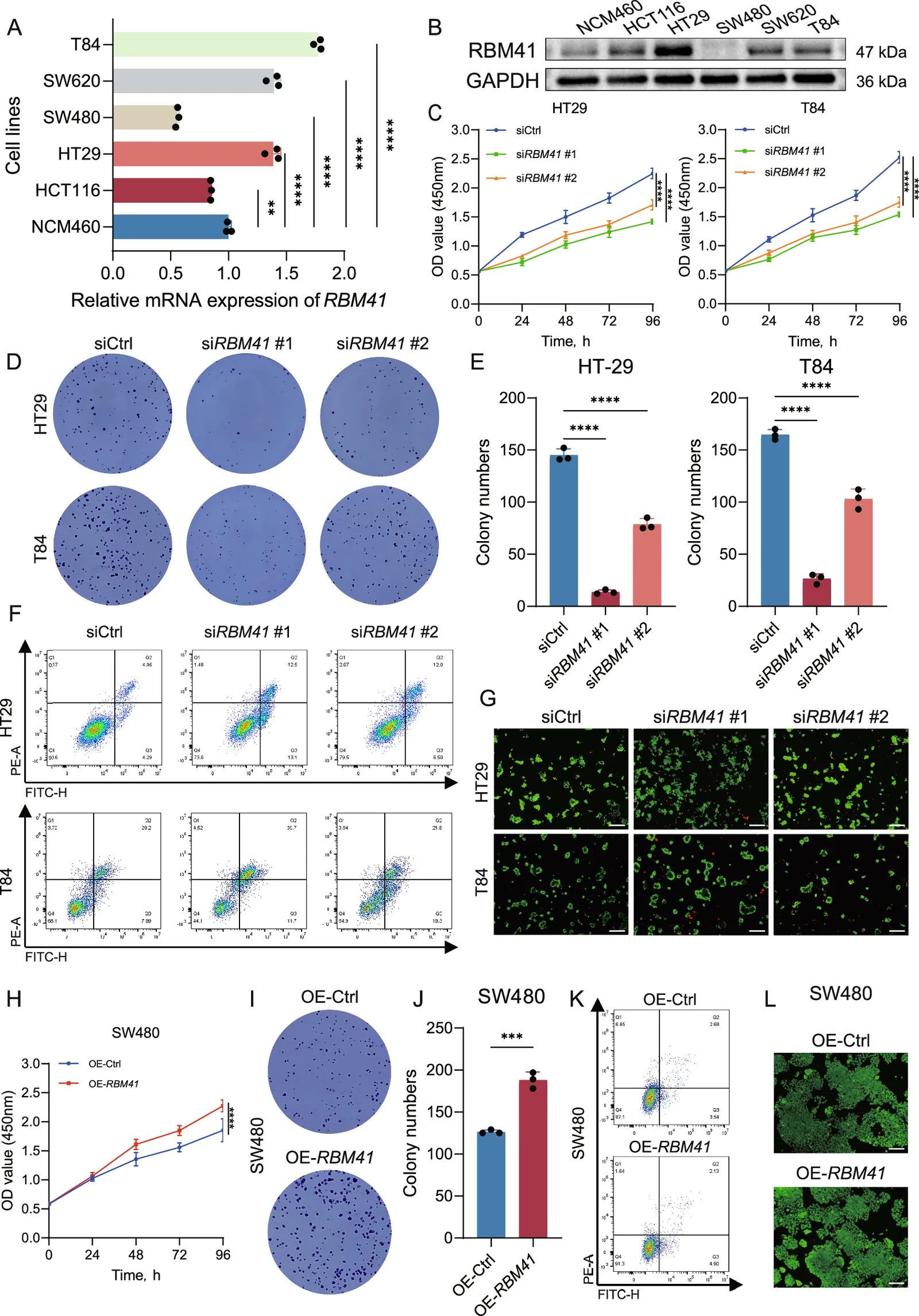
RBM41 promotes colorectal cancer cell proliferation and suppresses cell death. **A** Relative *RBM41* mRNA levels in a panel of human CRC cell lines (HCT116, HT29, SW480, SW620, T84) and a normal colonic epithelial cell line (NCM460), as determined by RT-qPCR. Data are presented as mean ± SD of *n* = 3 biological replicates. **B** Western blot analysis of RBM41 protein expression in the indicated CRC cell lines. GAPDH served as a loading control. The blots shown are representative of *n* = 3 biological replicates. **C** Cell proliferation assessed by CCK-8 assay in HT29 (left) and T84 (right) cells transfected with control siRNA (siCtrl) or two independent siRNAs targeting *RBM41* (si*RBM41* #1, #2). Data are presented as mean ± SD of *n* = 3 biological replicates. **D** Representative images of colony formation assays in HT29 (upper) and T84 (lower) cells after *RBM41* knockdown. The images shown are representative of *n* = 3 biological replicates. **E** Quantitative analysis of colony formation assays shown in (**D**). **F** Apoptosis analysis by flow cytometry using Annexin V-FITC/PI staining in HT29 (upper) and T84 (lower) cells after *RBM41* knockdown. The images shown are representative of *n* = 3 biological replicates. **G** Cell viability assessed by Live/Dead staining (Calcein-AM/PI; green, live cells; red, dead cells) in HT29 (upper) and T84 (lower) cells after *RBM41* knockdown. Scale bar, 200 μm. The images shown are representative of *n* = 3 biological replicates. **H** Cell proliferation was assessed by the CCK-8 assay in SW480 cells transfected with an empty vector (OE-Ctrl) or *RBM41* overexpression plasmid (OE-*RBM41*). Data are presented as mean ± SD of *n* = 3 biological replicates. **I** Representative images of colony formation assays in SW480 cells after *RBM41* overexpression. The data shown are representative of three independent experiments. The images shown are representative of *n* = 3 biological replicates. **J** Quantitative analysis of colony formation assays shown in (**I**). **K** Apoptosis analysis by flow cytometry in SW480 cells after *RBM41* overexpression. The images shown are representative of n = 3 biological replicates. **L** Cell viability assessed by Live/Dead staining (Calcein-AM/PI; green, live cells; red, dead cells) in SW480 cells after *RBM41* overexpression. Scale bar, 200 μm. The images shown are representative of *n* = 3 biological replicates. Statistical significance was determined using Student’s *t* test (**J**) or one-way ANOVA (**A**, **E**) or two-way ANOVA (**C**, **H**). Data are presented as mean ± SD. **, P < 0.01; ***, P < 0.001; ****, P < 0.0001.

Next, we assessed the expression profiles in a panel of CRC cell lines and found that *RBM41* was highly expressed in T84 and HT29 cells, whereas its expression was lowest in SW480 cells (Fig. 2A, B). HT29 and T84 cells were thus selected for the subsequent loss-of-function studies, whereas SW480 cells were selected for gain-of-function experiments to investigate the biological function of *RBM41* in CRC. Compared to control cells, knockdown of *RBM41* in HT29 and T84 cells significantly inhibited cell proliferation and viability (Fig. 2C) and clonogenic ability (Fig. 2D, E), while markedly increasing the proportion of apoptotic cells (Fig. 2F, G). Conversely, overexpression of *RBM41* in SW480 cells accelerated the cell proliferation rate, enhanced clonogenic ability, and improved cell survival (Fig. 2H–L). Collectively, these results demonstrate that *RBM41* promotes CRC proliferation and clonogenic activity while inhibiting the rate of cell apoptosis in vitro, suggesting its role as an oncogene in CRC.

### *RBM41* deficiency triggers synergistic lethality through concurrent apoptosis induction and autophagic flux blockade

**Fig. 3:**
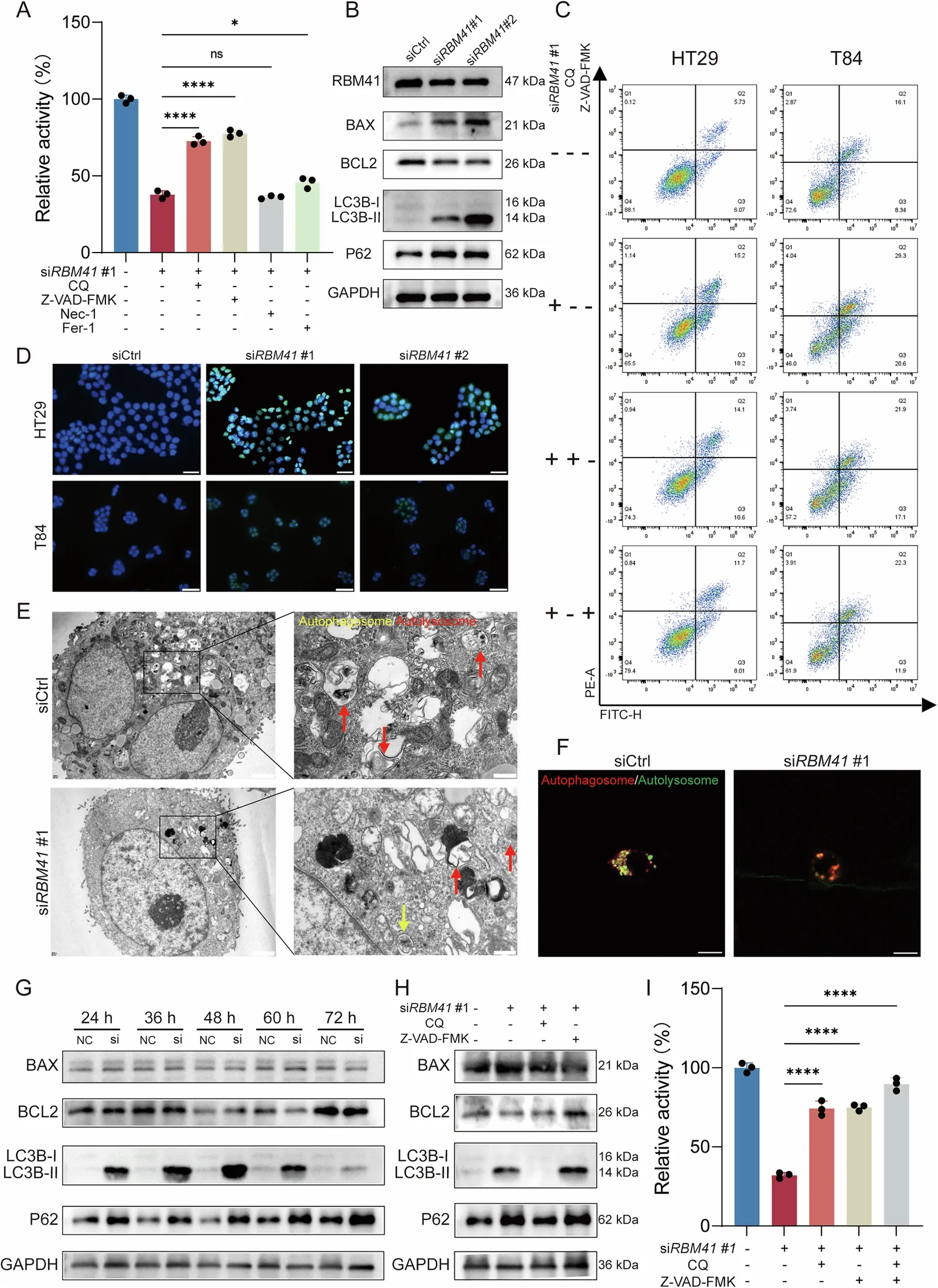
*RBM41* deficiency triggers synergistic lethality through concurrent apoptosis induction and autophagic flux blockade. **A** Cell viability measured by CCK 8 assay in HT29 cells transfected with siCtrl or si*RBM41* #1 and treated with inhibitors of autophagy (CQ), apoptosis (Z-VAD-FMK), necroptosis (Nec-1), or ferroptosis (Fer-1). Data are presented as mean ± SD of *n* = 3 biological replicates. **B** Western blot analysis of RBM41, apoptosis-related proteins (BAX, BCL2), and autophagy-related proteins (LC3B I, LC3B II, P62) in HT29 cells transfected with siCtrl or si*RBM41* (#1, #2). GAPDH served as a loading control. The blots shown are representative of *n* = 3 biological replicates. **C** Apoptosis analysis by flow cytometry in HT29 (left) and T84 (right) cells under the indicated conditions. The images shown are representative of *n* = 3 biological replicates. **D** TUNEL staining to detect DNA fragmentation in HT29 (upper) and T84 (lower) cells transfected with siCtrl or si*RBM41* (#1, #2). TUNEL-positive cells (green) and nuclei (blue) were counterstained with DAPI. Scale bar, 20 μm. The images shown are representative of *n* = 3 biological replicates. **E** Representative transmission electron microscopy (TEM) images of HT29 cells transfected with siCtrl or si*RBM41* #1. Red arrows indicate autophagosomes; yellow arrows indicate autolysosomes. Scale bar, 2 μm (left) and 500 nm (right). The images shown are representative of *n* = 3 biological replicates. **F** Assessment of autophagic flux by immunofluorescence staining using DALGreen (autophagosomes) and DAPRed (autolysosomes) probes in HT29 cells transfected with siCtrl or si*RBM41* #1. Scale bar, 10 μm. The images shown are representative of *n* = 3 biological replicates. **G** Western blot analysis of apoptosis and autophagy markers at the indicated time points (24–72 h) after transfection with NC (siCtrl) or si (si*RBM41* #1) in HT29 cells. The blots shown are representative of *n* = 3 biological replicates. **H** Western blot analysis of apoptosis and autophagy markers in HT29 cells under the indicated conditions. The blots shown are representative of *n* = 3 biological replicates. (**I** Cell viability measured by CCK-8 assay in HT29 cells under the indicated conditions Data are presented as mean ± SD of *n* = 3 biological replicates. Data shown in (**B**, **G**, **H**) are representative of at least three independent experiments. Statistical significance was determined using one-way ANOVA (**A**, **I**). Data are presented as mean ± SD. *, P < 0.05; ****, P < 0.0001; ns, not significant.

To determine how *RBM41* affects cell death in CRC, we first screened potential death pathways with pharmacological inhibitors. Treatment with either the autophagy inhibitor chloroquine (CQ) or the apoptosis inhibitor Z-VAD-FMK significantly rescued the decreased cell viability caused by *RBM41* knockdown, whereas inhibitors of ferroptosis or necroptosis had minimal effects (Fig. 3A). We next examined molecular changes associated with these pathways. *RBM41* knockdown concurrently upregulated the Bax while downregulating the BCL2; it also increased the LC3B-II/LC3B-I ratio along with P62 accumulation, confirming the activation of apoptosis and the impairment of autophagic flux in *RBM41*-deletion HT29 cells (Fig. 3B). Flow cytometry and TUNEL staining further corroborated the increased cell death and enhanced DNA fragmentation in HT29 cells following *RBM41* deletion, respectively (Fig. 3C, D). To further characterize the autophagic disruption, we performed transmission electron microscopy (TEM) and autophagy flux assays. Abundant autophagosome accumulation rather than autolysosomes was confirmed in *RBM41*-deleted HT29 cells (Fig. 3E). In line with the changes of key autophagic proteins, lysosome-sensitive fluorescent probes confirmed impaired autophagosome-lysosome fusion, collectively demonstrating the late-stage autophagic flux disruption following *RBM41* deletion (Fig. 3F). Interestingly, we found that changes in autophagy-related markers (LC3B and P62) occurred prior to alterations in apoptosis-related proteins (Bax and BCL2), indicating that autophagy precedes subsequent apoptosis upon *RBM41* deletion (Fig. 3G). However, inhibiting autophagy did not reverse the changes of Bax and BCL2 caused by *RBM41* knockdown; conversely, apoptosis inhibition did not alter autophagy markers in *RBM41*-deletion HT29 cells (Fig. 3H). Functionally, only simultaneous inhibition of both autophagy and apoptosis restored cell viability to the greatest extent (Fig. 3I), indicating that they act synergistically to mediate cell death induced by *RBM41* deficiency. Together, these results demonstrate that the apoptosis and disrupted autophagy resulting from *RBM41* loss are not mutually requisite but function through independent mechanisms to exert a synergistic lethal effect in CRC cells.

### *RBM41* suppresses *NDRG1* by impeding its pre-mRNA maturation

**Fig. 4:**
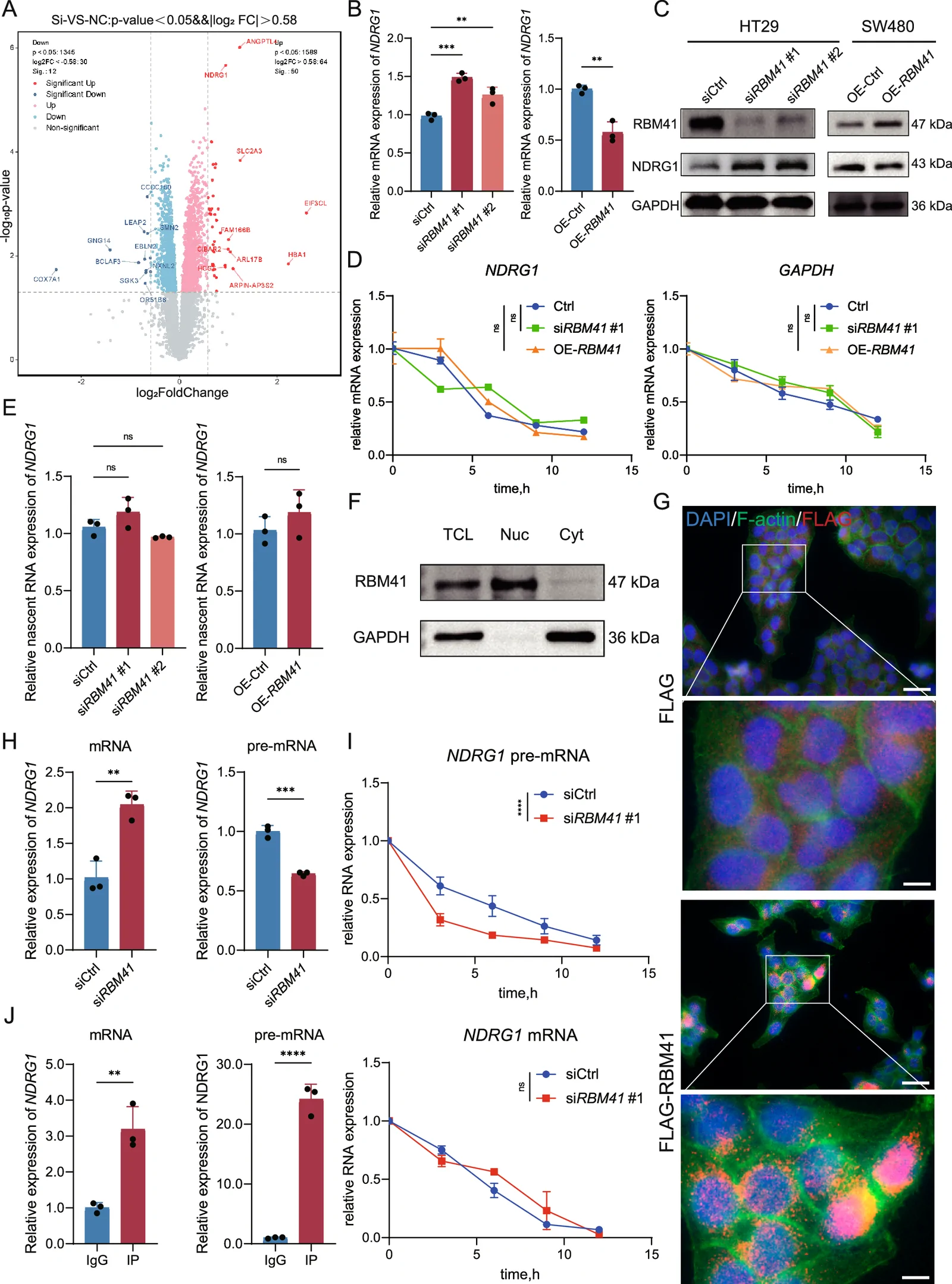
RBM41 suppresses *NDRG1* by impeding its pre-mRNA maturation. **A** Volcano plot of transcriptome sequencing (RNA seq) analysis in HT29 cells transfected with siCtrl (*n* = 3 biological replicates) or si*RBM41* #1 (*n* = 3 biological replicates). **B** Relative *NDRG1* mRNA levels measured by RT-qPCR in HT29 cells with *RBM41* knockdown (left) and SW480 cells with *RBM41* overexpression (right). Data are presented as mean ± SD of *n* = 3 biological replicates. **C** Western blot analysis of NDRG1 protein levels in HT29 cells with RBM41 knockdown (left) and SW480 cells with *RBM41* overexpression (right). GAPDH served as a loading control. The blots shown are representative of *n* = 3 biological replicates. **D** mRNA stability assay for *NDRG1* (left) and GAPDH (right) transcripts in HT29 cells treated with actinomycin D (5 μg/mL) under the indicated conditions. Data are presented as mean ± SD of *n* = 3 biological replicates. **E** Levels of nascent NDRG1 RNA (biotin-EU pull-down & RT-qPCR) with RBM41 knockdown (left) or overexpression (right). Data are presented as mean ± SD of *n* = 3 biological replicates. **F** Western blot analysis of RBM41 protein levels in total cell lysates (TCL), nuclear fractions (Nuc), and cytoplasmic fractions (Cyt) of HT29 cells. GAPDH was used as a cytoplasmic marker. The blots shown are representative of *n* = 3 biological replicates. **G** Subcellular localization of FLAG-RBM41 (red) by immunofluorescence. F-actin (green), DAPI (blue). Scale bars: 20 μm and 5 μm (inset). The images shown are representative of *n* = 3 biological replicates. (H) Relative levels of mature *NDRG1* mRNA (left) and pre-mRNA (right) measured by RT-qPCR in HT29 cells transfected with siCtrl or si*RBM41* #1. Data are presented as mean ± SD of *n* = 3 biological replicates. **I** Stability assay for *NDRG1* pre mRNA (upper) and mature mRNA (lower) in HT29 cells treated with actinomycin D after transfection with siCtrl or si*RBM41* #1. Data are presented as mean ± SD of *n* = 3 biological replicates. **J** RNA immunoprecipitation (RIP) assay using control IgG or anti RBM41 antibody, followed by RT-qPCR analysis of *NDRG1* mature mRNA (left) and pre-mRNA (right) levels. Data are presented as mean ± SD of *n* = 3 biological replicates. Statistical significance was determined using one-way ANOVA (**B** left, **E** left) or Student’s *t* test (**B** right, **E** right, **H**, **J**) or two-way ANOVA (**D**, **I**). Data are presented as mean ± SD. **, P < 0.01; ***, P < 0.001; ****, P < 0.0001; ns, not significant.

To elucidate the downstream molecular mechanisms by which *RBM41* regulates the malignant phenotype of CRC, transcriptome sequencing on *RBM41*-knockout cells was performed. The results revealed significant alterations in the expression of multiple genes following *RBM41* depletion. Among these, *NDRG1* was markedly upregulated and selected for further investigation due to its established role as a tumor suppressor in CRC [18–20] (Fig. 4A). We found that knockdown of *RBM41* increased both mRNA and protein levels of *NDRG1*, while *RBM41* overexpression decreased *NDRG1* expression in CRC cells (Fig. 4B, C), indicating that *RBM41* negatively regulates *NDRG1* expression. As an RNA-binding protein, *RBM41* likely exerts this regulation post-transcriptionally. However, actinomycin D assays showed *RBM41* depletion did not alter *NDRG1* mRNA stability (Fig. 4D), and quantification of nascent RNA revealed no effect on *NDRG1* transcription (Fig. 4E). Thus, we imply that *RBM41* regulates *NDRG1* through a mechanism distinct from controlling mRNA stability or transcription.

The subcellular localization of RBPs is closely related to their biological function and potential molecular roles [21]. We therefore investigated the subcellular localization of RBM41 and found it is predominantly localized in the nucleus in HT29 cells (Fig. 4F, 4G). As RBPs located in the nucleus are usually associated with post-transcriptional gene regulation [22], we hypothesized that *RBM41* might regulate *NDRG1* expression by modulating pre-mRNA processing. Indeed, *RBM41* knockdown led to a significant reduction in *NDRG1* pre-mRNA levels, accompanied by an increase in mature mRNA (Fig. 4H). Furthermore, stability assays demonstrated that the degradation rate of *NDRG1* pre-mRNA was significantly accelerated upon *RBM41* depletion (Fig. 4I), indicating that depletion of RBM41 relieves the block on pre-mRNA processing and accelerates its conversion to mature mRNA, thus demonstrating that RBM41 impedes this processing. To directly investigate the interaction between *RBM41* and *NDRG1* transcripts, we performed RNA immunoprecipitation (RIP) experiments. Our data confirmed that *RBM41* exhibits a stronger binding affinity for the pre-mRNA than for the mature transcript of *NDRG1* (Fig. 4J). These findings support that *RBM41* participates in pre-mRNA processing of *NDRG1* in CRC cells.

### Identification of a stem-loop structure in the 3′UTR of *NDRG1* pre-mRNA as the *RBM41*- binding site

**Fig. 5:**
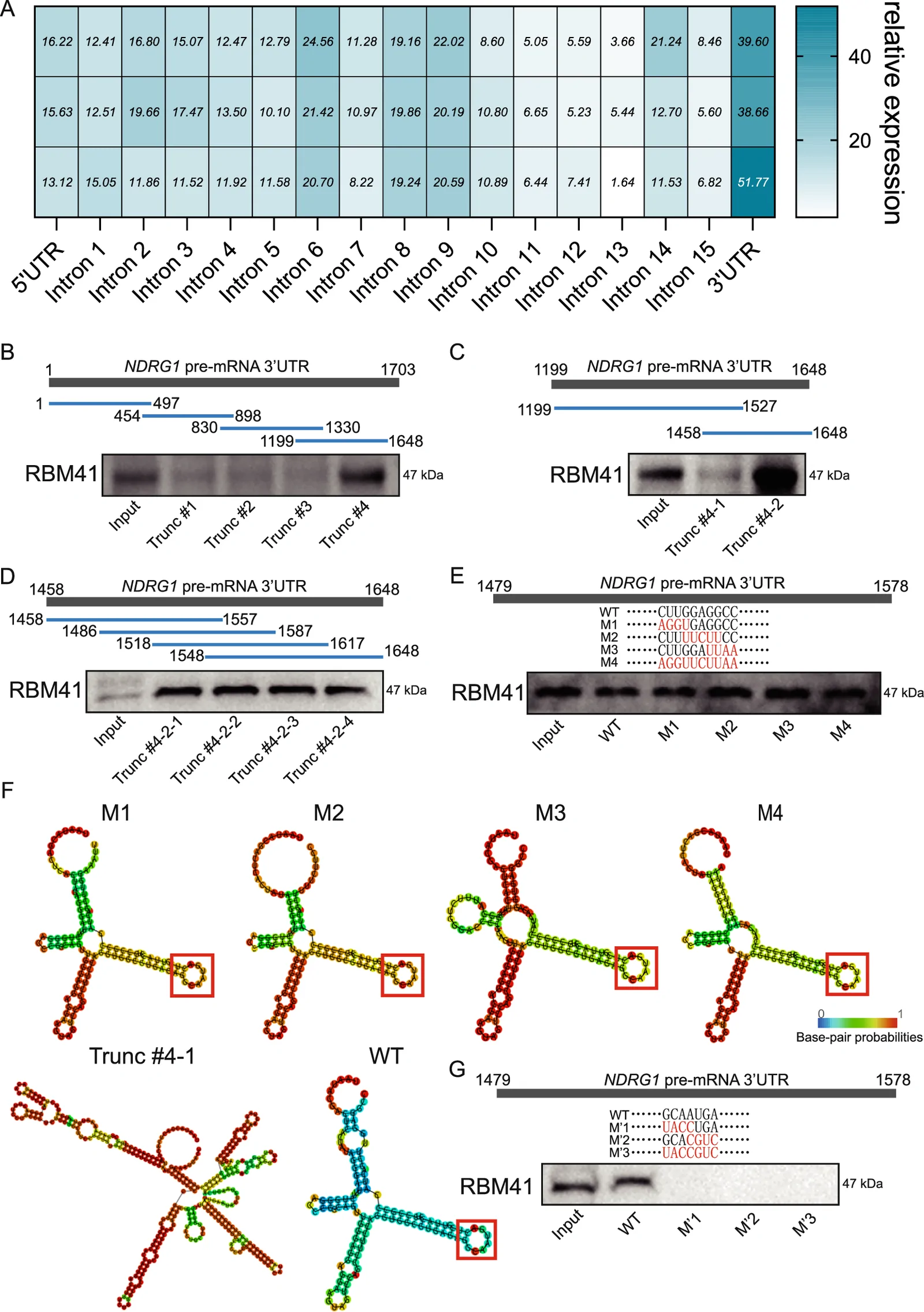
RBM41 binds to a stem-loop structure in the 3′UTR of *NDRG1* pre-mRNA. **A** RNA immunoprecipitation (RIP) assay followed by RT-qPCR analysis of the indicated regions (5’ UTR, Intron 1–15, and 3’ UTR) of *NDRG1* pre mRNA in HT29 cells transfected with FLAG-tagged RBM41, using control IgG or an anti-FLAG antibody. Data are presented as mean ± SD of *n* = 3 biological replicates. RNA pull-down assays using biotin-labeled fragments covering different regions of the *NDRG1* 3’ UTR: 1–497 nt, 454–898 nt, 830–1330 nt, and 1199–1648 nt (**B**); 1199–1527 nt and 1458–1648 nt (**C**); 1458–1557 nt, 1486–1587 nt, 1518–1617 nt, and 1548–1648 nt (**D**); and truncated variants of 1479–1578 nt (**E**). RBM41 protein bound to these RNA fragments was detected by Western blot. The blots shown are representative of *n* = 3 biological replicates. **F** Predicted secondary RNA structures of the key binding fragment and a non-binding control fragment, as obtained from the RNAfold web server (http://rna.tbi.univie.ac.at/). The conserved GCAAUGA motif within the stem-loop structure is boxed in red. **G** Western blot analysis of RBM41 binding to wild-type (WT) and mutant stem-loop RNA probes, in which the GCAAUGA motif was systematically disrupted. The blots shown are representative of *n* = 3 biological replicates. Data shown in (**B**–**E**, **G**) are representative of at least three independent experiments.

Since *RBM41* binds predominantly to the precursor rather than the mature mRNA of *NDRG1*, we hypothesized that *RBM41* directly binds to the non-coding regions of *NDRG1* pre-mRNAs. To test this, we performed RIP assay coupled to RT-qPCR analysis and revealed the highest enrichment in the 3’ UTR of *NDRG1* pre-mRNA (Fig. 5A). To further identify the *RBM41*-binding sequence in *NDRG1* pre-mRNA, we constructed a series of biotin-labeled RNA truncations spanning different segments of the *NDRG1* 3’ UTR. Pull-down experiments indicated that *RBM41* preferentially recognizes and binds within an approximately 120 nucleotides (nt) of the *NDRG1* 3’ UTR (Fig. 5B, C). Furthermore, we subdivided this 120 nt region into four segments. Unexpectedly, all subdivided fragments effectively pulled down *RBM41* protein, and mutants that were designed based on the overlapping sequence also retained binding ability (Figs. 5D, E), implying that the *RBM41*/*NDRG1* interaction might not depend on a specific short linear motif but rather on RNA secondary structure.

To verify this hypothesis, we predicted the secondary structures of the M1, M2, M3, M4, and wild-type (WT) fragments using bioinformatics tools (http://rna.tbi.univie.ac.at/) and compared them with the non-binding control fragment Trunc #4-1, which revealed that Trunc #4-1 lacked the GCAAUGA-containing stem-loop structure present in all *RBM41*-binding fragments (Fig. 5F). Thus, we designed three mutant variants in which the conserved GCAAUGA motif was systematically disrupted to explore the functional importance of this stem-loop structure. Interaction with *RBM41* proteins was detected in the pull-down experiments with the wild-type fragment, but not with those three mutants (Fig. 5G), indicating the binding between *RBM41* and the stem-loop structure in *NDRG1* pre-mRNA. These results demonstrate that *RBM41* recognizes and binds to a specific secondary structure characterized by a stem-loop containing the GCAAUGA motif within the 3′UTR of *NDRG1* pre-mRNA, thereby inhibiting the processing and maturation of the *NDRG1* pre-mRNA into functional mRNA.

### *NDRG1* is the key downstream effector mediating *RBM41*-regulated cell death

**Fig. 6:**
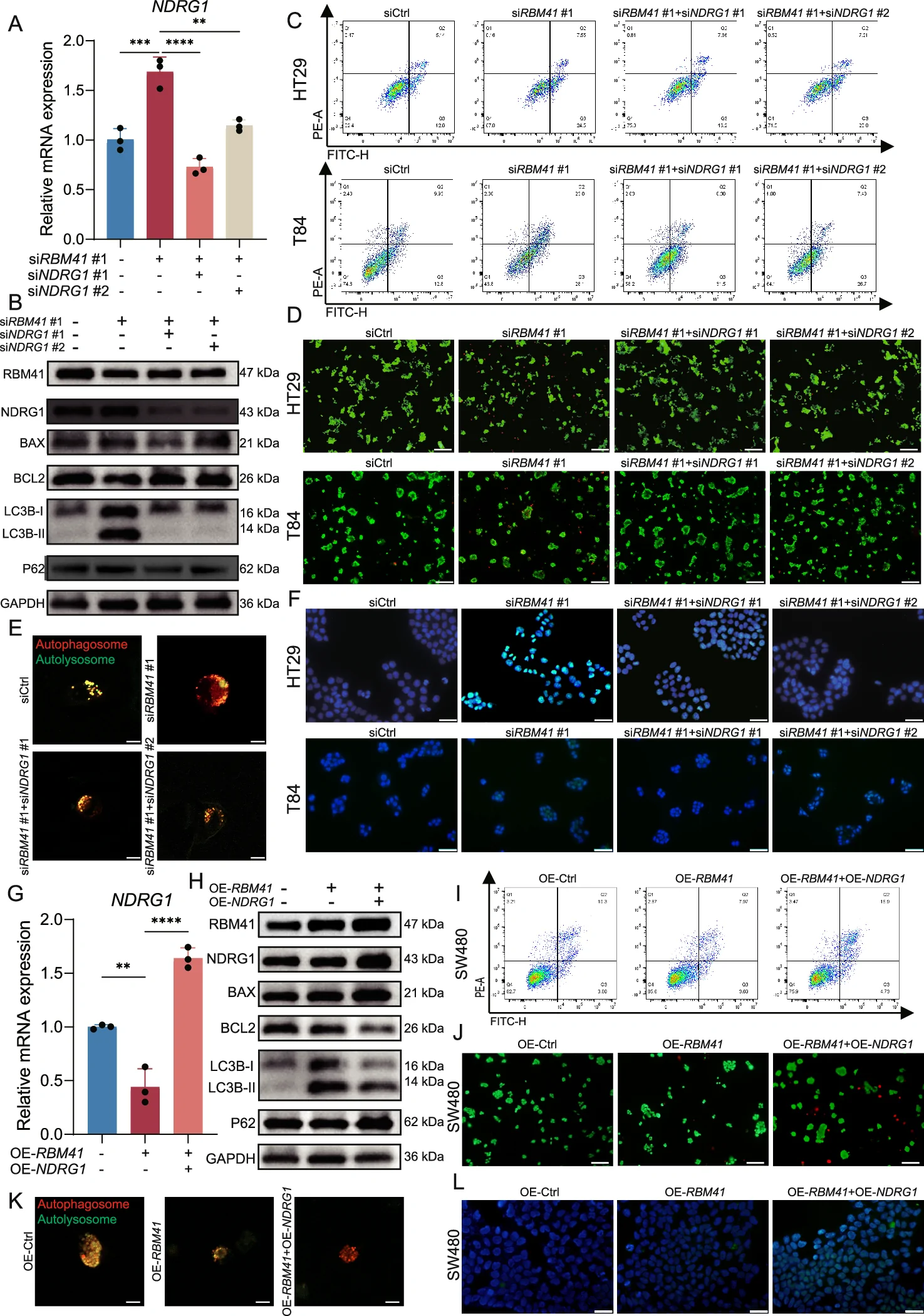
*NDRG1* is the key downstream effector mediating *RBM41*-regulated cell death. **A**–**F** HT29 cells were transfected with control siRNA (siCtrl), *RBM41*-targeting siRNA (si*RBM41* #1), or si*RBM41* #1 combined with two independent *NDRG1*-targeting siRNAs (si*RBM41* #1 + si*NDRG1* #1/#2). All experiments were performed with *n* = 3 biological replicates. (A) Cell viability measured by CCK-8 assay. **B** Western blot analysis of RBM41, NDRG1, apoptosis-related (BAX, BCL2), and autophagy-related (LC3B, P62) proteins. **C** Apoptosis analysis by flow cytometry. **D** Cell viability was assessed by Live/Dead staining (Calcein-AM/PI; green, live cells; red, dead cells). Scale bar, 200 μm. **E** Autophagic flux was assessed by immunofluorescence staining using DALGreen (autophagosomes) and DAPRed (autolysosomes) probes. Scale bar, 10 μm. **F** DNA fragmentation detected by TUNEL staining. TUNEL-positive cells (green) and nuclei (blue) were counterstained with DAPI. Scale bar, 20 μm. **G**–**L** SW480 cells were transfected with empty vector (OE-Ctrl), *RBM41* overexpression plasmid (OE-*RBM41*), or OE-*RBM41* combined with *NDRG1* overexpression plasmid (OE-*RBM41* + OE-*NDRG1*). All experiments were performed with *n* = 3 biological replicates. **G** Cell viability measured by CCK-8 assay. **H** Western blot analysis of RBM41 and NDRG1 protein levels. **I** Apoptosis analysis by flow cytometry. **J** Cell viability assessed by Live/Dead staining (Calcein-AM/PI; green, live cells; red, dead cells). Scale bar, 200 μm. **K** Autophagic flux was assessed by immunofluorescence staining using DALGreen (autophagosomes) and DAPRed (autolysosomes) probes. Scale bar, 10 μm. **L** DNA fragmentation detected by TUNEL staining. TUNEL-positive cells (green) and nuclei (blue) were counterstained with DAPI. Scale bar, 20 μm. Data shown in (**B**, **H**) are representative of at least three independent experiments. Statistical significance was determined using one-way ANOVA (**A**, **G**). Data are presented as mean ± SD. **, P < 0.01; ***, P < 0.001; ****, P < 0.0001.

To clarify the role of *NDRG1* in the *RBM41*-regulated functional effects in CRC, we performed a series of rescue experiments. Simultaneous knockdown of *NDRG1* in *RBM41*-deletion CRC cells effectively reversed the upregulation of *NDRG1* expression caused by *RBM41* deficiency (Fig. 6A). Western blot results showed that restoring *NDRG1* expression significantly ameliorated the aberrant expression of apoptosis-related proteins and autophagy-related markers induced by *RBM41* knockdown (Fig. 6B). *NDRG1* deletion markedly alleviated the increase in cell death induced by *RBM41* knockdown, and the restored cell survival rate was evidenced by consistent results from both flow cytometric analysis (Fig. 6C) and live/dead staining (Fig. 6D). We also observed a significant rescue of apoptosis and of impaired autophagic flux in RBM41-underexpressing CRC cells when simultaneously knocking down NDRG1 (Fig. 6E, F). Conversely, overexpression of *NDRG1* counteracted the pro-survival effects of *RBM41* in *RBM41*-overexpressing SW480 cells (Fig. 6G-L). These results consistently demonstrate that *NDRG1* is a key downstream effector molecule through which *RBM41* regulates both autophagy and apoptosis, thereby mediating its oncogenic functions in CRC.

### In vivo experiments confirm that *RBM41* promotes CRC growth by regulating *NDRG1*

**Fig. 7:**
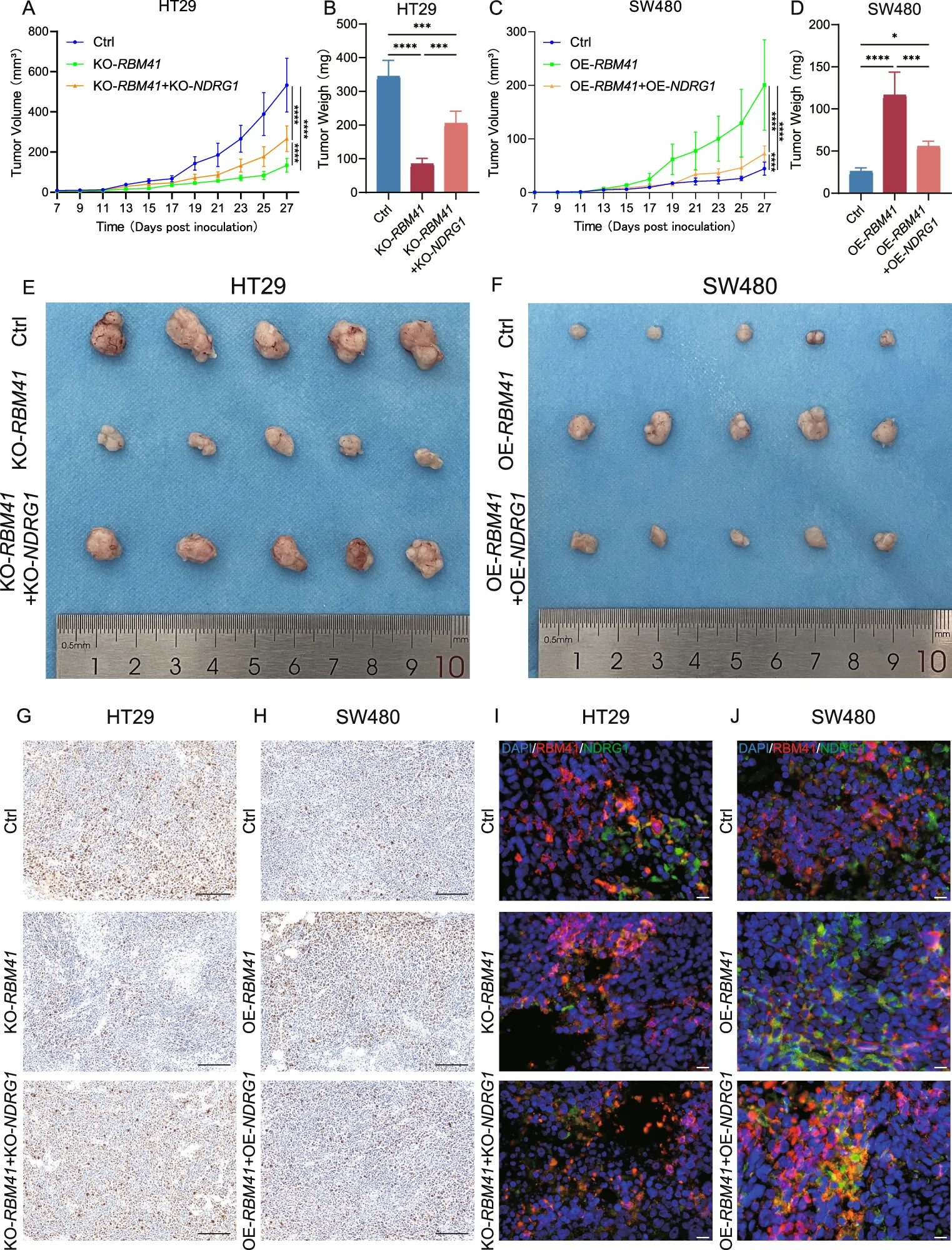
*RBM41* promotes colorectal tumor growth by regulating *NDRG1* in vivo. **A** Growth curves of HT29-derived xenograft tumors in nude mice from the following groups: control (Ctrl), *RBM41* knockdown (KO-*RBM41*), and *RBM41* knockdown combined with *NDRG1* knockdown (KO-*RBM41* + KO-*NDRG1*). Tumor volumes were measured at the indicated time points (7–27 days). Data are presented as mean ± SD of *n* = 5 biological replicates. **B** Tumor weights of HT29-derived xenografts from the Ctrl, KO-*RBM41*, and KO-*RBM41* + KO-*NDRG1* groups on day 27. Data are presented as mean ± SD of *n* = 5 biological replicates. **C** Growth curves of SW480-derived xenograft tumors in nude mice from the following groups: control (Ctrl), *RBM41* overexpression (OE-*RBM41*), and *RBM41* overexpression combined with *NDRG1* overexpression (OE-*RBM41* + OE-*NDRG1*). Data are presented as mean ± SD of *n* = 5 biological replicates. **D** Tumor weights of SW480-derived xenografts from the Ctrl, OE-*RBM41*, and OE-*RBM41* + OE-*NDRG1* groups on day 27. Data are presented as mean ± SD of *n* = 5 biological replicates. **E** Representative images of HT29-derived tumors from the Ctrl, KO-*RBM41*, and KO-*RBM41* + KO-*NDRG1* groups. The images shown are tumors from *n* = 5 biological replicates. **F** Representative images of SW480-derived tumors from the Ctrl, OE-*RBM41*, and OE-*RBM41* + OE-*NDRG1* groups. The images shown are tumors from n = 5 biological replicates. **G** Immunohistochemical staining of Ki-67 in HT29-derived tumor sections from the Ctrl, KO-*RBM41*, and KO-*RBM41* + KO-*NDRG1* groups. Scale bar, 100 μm. The images shown are representative of *n* = 5 biological replicates. **H** Immunohistochemical staining of Ki-67 in SW480-derived tumor sections from the Ctrl, OE-*RBM41*, and OE-*RBM41* + OE-*NDRG1* groups. Scale bar, 100 μm. The images shown are representative of *n* = 5 biological replicates. **I** Immunofluorescence staining of *RBM41* (red) and *NDRG1* (green) in HT29-derived tumor sections from the Ctrl, KO-*RBM41*, and KO-*RBM41* + KO-*NDRG1* groups. Nuclei were counterstained with DAPI (blue). Scale bar, 20 μm. The images shown are representative of *n* = 5 biological replicates. **J** Immunofluorescence staining of *RBM41* (red) and *NDRG1* (green) in SW480-derived tumor sections from the Ctrl, OE-*RBM41*, and OE-*RBM41* + OE-*NDRG1* groups. Nuclei were counterstained with DAPI (blue). Scale bar, 20 μm. The images shown are representative of *n* = 5 biological replicates. Statistical significance was determined by one-way ANOVA (**B**, **D**) or two-way ANOVA (**A**, **C**). Data are presented as mean ± SD.*, P < 0.05; ***, P < 0.001; ****, P < 0.0001.

To validate the anticancer effects of the *RBM41*-*NDRG1* axis in vivo, we established xenograft tumor models in nude mice. In the HT29 cell xenograft model, the *RBM41*-knockout group showed significantly slowed tumor growth, as evidenced by markedly reduced tumor volume and weight at day 27 compared to the control group. Importantly, this tumor growth inhibitory effect by *RBM41* depletion was significantly reversed when *NDRG1* was simultaneously knocked out (Fig. 7A, B, E). Conversely, overexpression of *RBM41* markedly promoted tumor growth, which was effectively counteracted by co-overexpression of *NDRG1* in the SW480 xenograft model (Fig. 7C, D, 7F). Consistently, Ki-67 immunohistochemistry showed that *RBM41* depletion suppressed tumor cell proliferation, and this effect was reversed by simultaneous *NDRG1* knockout (Fig. 7G, H). Notably, immunofluorescence co-staining further supported our statement that the tumor areas with the highest proliferative activity were precisely those exhibiting high RBM41 and low NDRG1 expression (Fig. 7I, J). It should be noted that the cytoplasmic signal can be attributed to the subcellular site of protein synthesis and processing, as well as to potential non-specific binding of the polyclonal antibody. Collectively, these in vivo results demonstrate that RBM41 drives colorectal tumor growth through the regulation of *NDRG1*.

### *RBM41* expression level predicts patient prognosis and PDO drug sensitivity

**Fig. 8:**
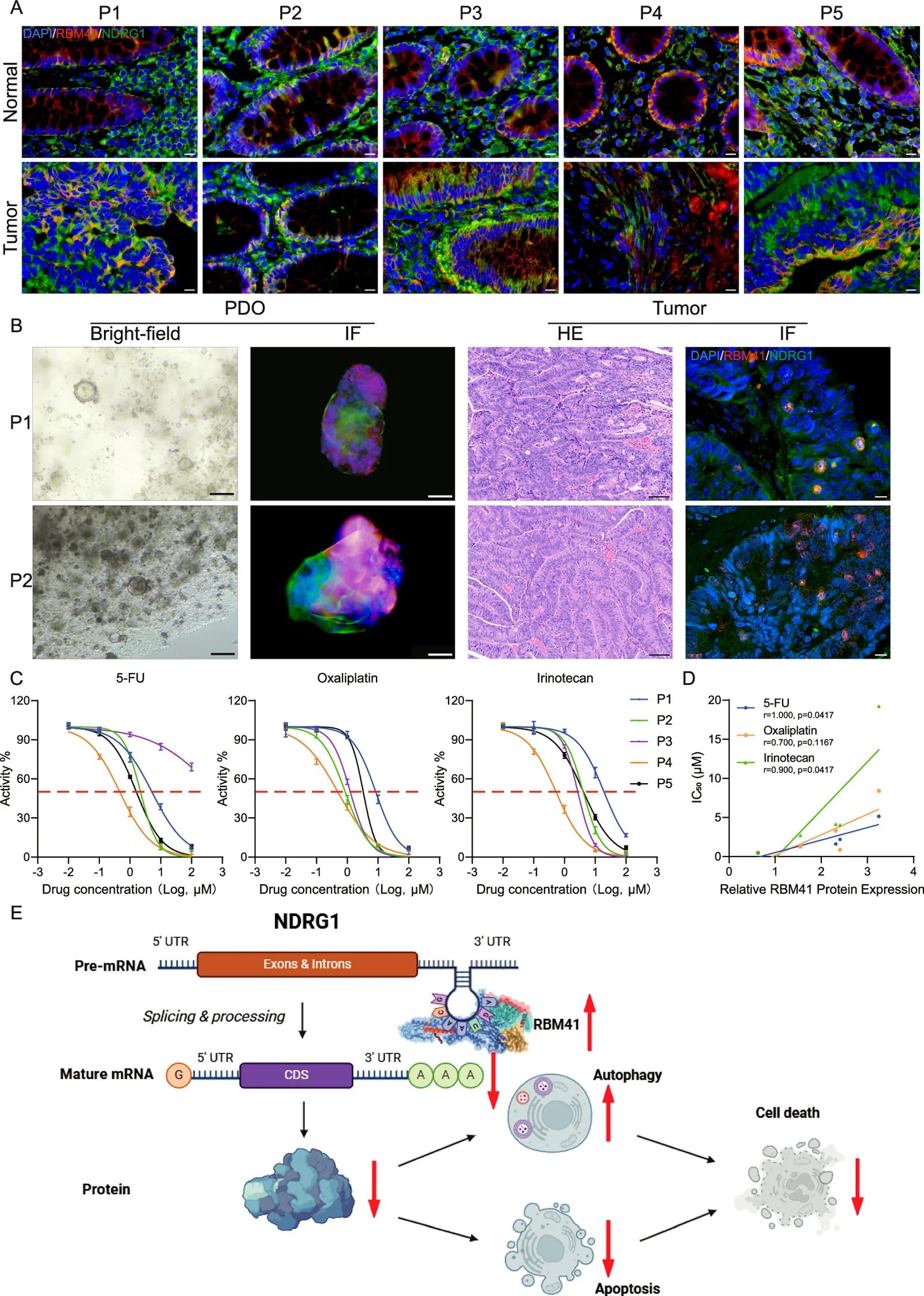
RBM41 expression correlates with chemoresistance in colorectal cancer and patient-derived organoids. **A** Immunofluorescence staining of RBM41 (red) and NDRG1 (green) in five representative pairs of CRC and matched adjacent normal tissues. Nuclei were counterstained with DAPI (blue). Scale bar, 100 μm. The images shown are representative of *n* = 3 biological replicates. **B** Characterization of PDOs. From left to right: bright-field images of PDO structures (scale bar, 200 μm); immunofluorescence staining of RBM41 (red) and NDRG1 (green) in PDOs (scale bar, 20 μm); H&E staining of primary tumor tissues (scale bar, 100 μm); and immunofluorescence staining of RBM41 and NDRG1 in corresponding tumor sections (scale bar, 20 μm). Nuclei were stained with DAPI (blue). The images shown are representative of *n* = 5 biological replicates. **C** IC₅₀ values of first-line chemotherapeutic agents (5-fluorouracil, oxaliplatin, and irinotecan) in PDOs derived from five CRC patients, determined by ATP-based viability assays. The horizontal dashed line represents 50% inhibition. Data are presented as mean ± SD of *n* = 3 biological replicates. **D** Spearman correlation analysis between RBM41 protein expression levels (quantified by grayscale analysis) in tumors from five patients and IC₅₀ values of chemotherapeutic agents. RBM41 expression positively correlates with IC₅₀ values. **E** Schematic model illustrating that RBM41 binds to a specific site in the 3′UTR of *NDRG1* pre-mRNA, thereby inhibiting its maturation; targeting this *RBM41*/*NDRG1* axis induces autophagic cell death and apoptosis in CRC.

Then, we investigated the expression of RBM41 and NDRG1 in tumors and matched normal tissues from five CRC patients. In patient tumors, high *RBM41* generally associates with low *NDRG1* (Fig. 8A), providing direct clinical support for their regulatory relationship in CRC. PDOs are recognized as a superior ex vivo platform for investigating chemotherapy resistance mechanisms, as they can recapitulate the molecular heterogeneity, pathological features, and drug response profiles of the primary tumors [23–25]. Our established PDOs retained the molecular characteristics of the parental tumors, especially the negative correlation between *RBM41* and *NDRG1* expression (Fig. 8B). To investigate the potential of *RBM41* as a predictor of chemotherapy response, we treated the organoids with three first-line clinical chemotherapeutic agents and measured cell viability via adenosine triphosphate (ATP) activity assays. PDOs with high RBM41 expression had significantly higher half-maximal inhibitory concentration (IC₅₀) for various chemotherapeutic agents compared to those with low RBM41 expression (Fig. 8C). Moreover, correlation analysis could also support that RBM41 expression levels positively correlated with IC₅₀ values for clinical first-line chemotherapy regimens (Fig. 8D), indicating that elevated RBM41 expression was associated with increased chemoresistance in CRC. These results collectively indicate that *RBM41* expression is a biomarker for malignant proliferation but also for drug resistance to standard chemotherapies, highlighting its potential value as a prognostic molecule and therapeutic target in CRC.

## Discussion

RBPs function as pivotal post-transcriptional orchestrators of cancer progression [26–28]. Among them, the RBM protein family is known for its diverse functions by regulating RNA processing [29–31]. Although *RBM41* has been shown to play a crucial role in regulating mRNA processing [17], its specific role in colorectal carcinogenesis remains largely undefined. In this study, we demonstrated that *RBM41* is significantly overexpressed in CRC and is associated with an unfavorable prognosis, suggesting its potential value as a prognostic biomarker. Functionally, knockdown of *RBM41* synergistically triggers obstruction of autophagic flux and activation of the apoptotic pathway in CRC cells, underscoring its central role in maintaining cellular homeostasis. Importantly, we observed that upon knockout of *RBM41*, CRC cells first exhibit inhibited autophagic flux, followed by apoptosis. This is consistent with previous reports [32] that autophagy, as a homeostatic maintenance mechanism, occurs rapidly upon cellular stress responses to promote cell survival by clearing damaged components. When the autophagic process is obstructed or fails, the accumulated damage signals subsequently trigger the irreversible apoptotic program. It is noteworthy that the critical role of *RBM41* in coordinating cell fate does not exist in isolation but constitutes an important component of the complex regulatory network of the RBM protein family. Similar to our findings, RBM22 induces apoptosis by regulating c-Myc pathway [33], whereas RBM33 [34] and RBM45 [35] regulate autophagy through distinct mechanisms to suppress cell death. In in vivo tumor models, targeting *RBM41* significantly inhibited tumor growth and reduced tumor volume, further verifing the tumor-promoting role of *RBM41*. Drug sensitivity assays revealed that organoids with high *RBM41* expression exhibited stronger resistance to multiple first-line chemotherapeutic agents, indicating that *RBM41* is not only an oncogene driving tumor proliferation but also a key mediator of chemoresistance. These data provide a theoretical basis for subsequent CRC treatment and overcoming chemotherapy resistance.

Furthermore, we have identified *NDRG1* as a key factor through which *RBM41* participates in regulating the progression of CRC. NDRG1 has been demonstrated to function as a tumor suppressor in colorectal cancer [36]. Overexpression of *NDRG1* significantly inhibits cancer cell proliferation and invasion, accompanied by increased apoptosis [18]. In this study, consistent results from both in vitro and in vivo experiments showed that knockdown of *NDRG1* effectively reverses the tumor-suppressive effects induced by *RBM41* deficiency. This finding aligns with the pro-tumorigenic phenotype observed upon *RBM41* overexpression. It provides key evidence that *RBM41* drives tumorigenesis through the negative regulation of *NDRG1*. Analysis of clinical samples shows a significant negative correlation between *RBM41* and *NDRG1* levels at both the protein and mRNA levels in tumor tissues, which was successfully replicated in PDOs. These findings provide strong clinical evidence for the oncogenic driver role of the *RBM41*-*NDRG1* axis.

Our work also revealed a novel mechanism distinct from most RBM family members, clarifying that *RBM41* promotes CRC progression by impeding the maturation of *NDRG1* pre-mRNA. Specifically, *RBM41*, which is predominantly localized in the nucleus, does not affect the transcription or degradation of *NDRG1* mRNA but exerts its oncogenic role by binding to a stem-loop structure containing a “GCAAUGA” motif within the 3’ UTR of *NDRG1* pre-mRNA to inhibit its proper maturation. This leads to post-transcriptional dysregulation of the key tumor suppressor *NDRG1*, ultimately driving malignant tumor progression (Fig. 8E). RNA secondary structures act as dynamic conformational switches, finely regulating RNA fate by masking or exposing key regulatory elements [37]. This study demonstrates that *RBM41* binds to the stem-loop structure of *NDRG1* pre-mRNA, likely interfering with its normal processing through steric hindrance or the recruitment of specific co-factors [38]. This interaction is proposed to ultimately reduce the abundance of functional mature mRNA, possibly by hindering the assembly of the spliceosome complex or interfering with the polyadenylation process. The higher specificity of RNA structure-based regulatory mode compared to simple linear sequence recognition enables *RBM41* to precisely select specific targets. The possibility that *RBM41* recognizes multiple RNA motifs is not excluded and needs further exploration.

Although this study has achieved the important advances described above, several limitations should be acknowledged. First, the upstream regulatory mechanisms responsible for the aberrantly high expression of RBM41 in colorectal cancer remain poorly understood, and the factors or signaling pathways contributing to its overexpression require further investigation. Second, as an RNA-binding protein, the precise RNA-binding domain of RBM41 has not been fully elucidated, and although it may recognize multiple RNA motifs, only one such motif was identified in this study. Third, the clinical sample size collected in this study is relatively small, which may limit the statistical power and generalizability of the observed negative correlation between RBM41 and NDRG1.

To address these limitations, future studies should be conducted from the following perspectives: fine mapping of the RNA-binding domain of RBM41 and comprehensive identification of its recognized RNA target motifs using high-throughput techniques such as CLIP-seq; large-scale, multicenter cohort studies to fully validate the clinical significance of the negative correlation between RBM41 and NDRG1 and its prognostic value in colorectal cancer, thereby overcoming the limitations imposed by the current small sample size; and in-depth investigation of the upstream molecular mechanisms governing the aberrant expression of RBM41, including transcriptional, epigenetic, or post-transcriptional regulation, such as identifying upstream transcription factors, non-coding RNAs, or RNA modification‑associated proteins, with the goal of fully elucidating the regulatory network of RBM41 in colorectal cancer development and progression.

In summary, this study identifies a new oncogenic RBM protein and reveals a novel oncogenic mechanism based on the recognition of RNA secondary structures to regulate pre-mRNA processing. Our work indicates that *RBM41* recognizes a specific stem-loop structure in the *NDRG1* pre-mRNA 3’ UTR, inhibiting its maturation. The inhibition of the tumor suppressor *NDRG1* pathway by *RBM41* thereby drives malignant progression and chemotherapy resistance in CRC. These findings provide a new theoretical foundation and potential target for the molecular subtyping and targeted therapy of CRC.

## Conclusions

This study reveals that RBM41 drives CRC progression and chemoresistance by directly binding to the 3’ UTR of the *NDRG1* pre-mRNA and inhibiting its maturation. These findings establish RBM41 as a crucial post-transcriptional regulator and a potential prognostic biomarker, highlighting the therapeutic potential of targeting the *RBM41-NDRG1* axis in CRC.

## Materials and methods

### Cell lines and cell culture

The NCM460, HCT116, HT29, SW480, SW620, and T84 cell lines were obtained from Servicebio (Wuhan, China) and authenticated by short tandem repeat (STR) profiling. NCM460 cells were cultured in RPMI 1640 medium (Meilunbio, MA0215) supplemented with 10% fetal bovine serum (FBS, Meilunbio, PWL001) and 1% penicillin/streptomycin (Meilunbio, MA0110). HCT116 and HT29 cells were maintained in DMEM (Meilunbio, MA0212) containing 10% FBS and 1% penicillin/streptomycin. SW480 and SW620 cells were grown in L-15 medium (Meilunbio, MA0547) with 10% FBS and 1% penicillin/streptomycin. T84 cells were cultured in DMEM/F12 (Meilunbio, MA0214) supplemented with 10% FBS and 1% penicillin/streptomycin. All cell lines were maintained in a humidified incubator at 37°C with 5% CO₂ and routinely tested for mycoplasma contamination via PCR.

For more detailed information regarding the materials and methods used, please refer to the full experimental procedures in the Supplementary Materials and Methods.

### Ethics approval and consent to participate

This study strictly adhered to the relevant ethical guidelines. The acquisition and use of human tissue samples and related clinical data were approved by the Ethics Committee of The First Hospital of Jilin University (Approval No.: 24K151-001), and informed consent was obtained from the patients. All animal experimental procedures complied with international animal welfare guidelines and were approved by the Animal Welfare and Research Ethics Committee at Jilin University (Approval No.: SY202507007).

## Supplementary information

Supplementary Materials and Methods

Full and Uncropped Western Blots

Supplementary Tables

## Data Availability

The data that support the findings of this study are available from the corresponding author upon reasonable request.
