# Correction: RNA binding motif protein RBM41 promotes colorectal tumorigenesis by impeding the maturation of *NDRG1* pre-mRNA

**DOI:** 10.1038/s41420-026-03341-2

**Published:** 2026-10-13

**Authors:** Yanxin Liu, Jianfeng Mu, Jiming Yu, Qirong Li, Tiantian Li, Qiang Feng, Tong Sun, Ke Sun, Yang Hao, Jinhai Yu, Dongxu Wang

**Affiliations:** 1 Department of Gastric and Colorectal Surgery, General Surgery Center, The First Hospital of Jilin University, Changchun, PR China; 2 Department of Laboratory Animals, College of Animal Sciences, Jilin University, Changchun, PR China

**Keywords:** Cancer, Molecular biology

Correction to: *Cell Death Discovery* https://doi.org/10.1038/s41420-026-03197-6, published online 20 June 2026

Since the online publication of this article, the authors have noticed that there was an error in Fig. 3. In the original version of this article, an error occurred during figure assembly. Due to a copy-paste mistake, the TUNEL staining image of the HT29 cell line with si*RBM41* #1 in Figure 3D was inadvertently misplaced during figure assembly and upload, and the correct original image was not presented. The accurate Figure 3D has now been replaced, as shown in the Amended Figure 3.

Furthermore, in Figure 3A, the abbreviation for necroptosis should be Nec-1 instead of Ncr-1, which we have modified in the Amended Figure 3. This correction does not affect the results or conclusions of the study. We apologize for any inconvenience caused.

**Original Figure 3**

**Figure:**
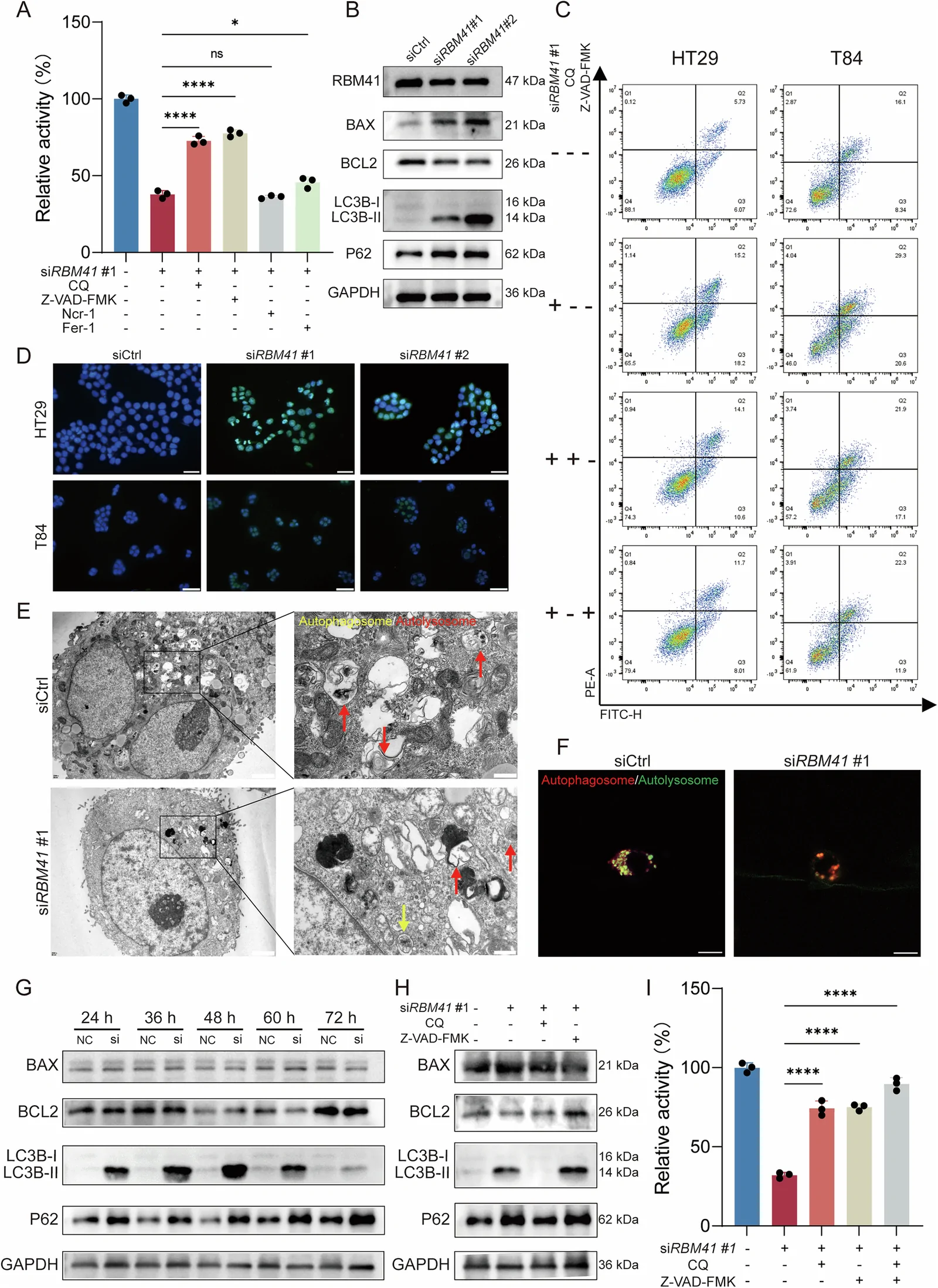

**Amended Figure 3**

**Figure:**
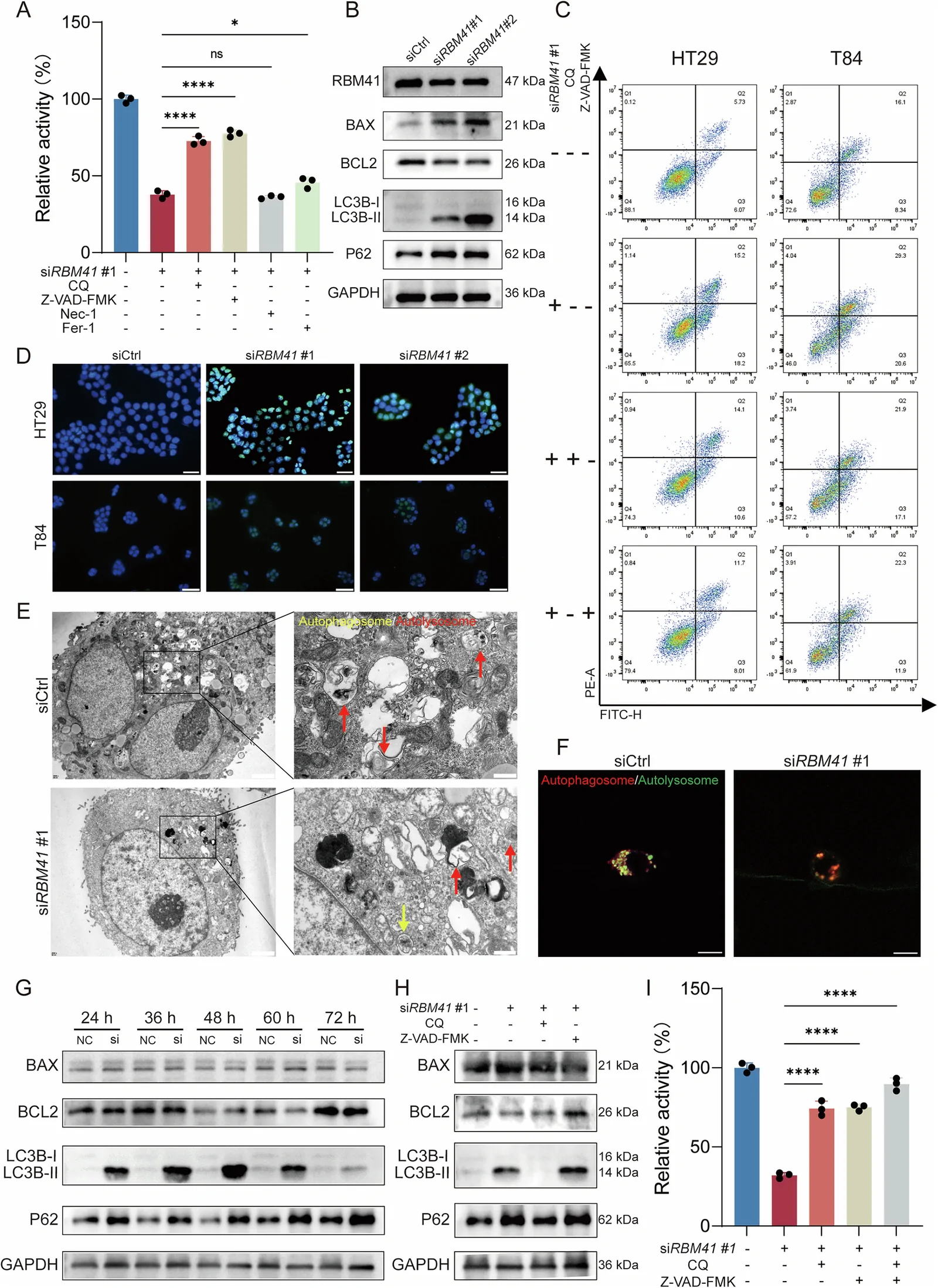

The original article has been corrected.

