# Supplementary material for: RNA binding motif protein RBM41 promotes colorectal tumorigenesis by impeding the maturation of NDRG1 pre-mRNA
Source: Cell Death Discov. 2026 Jun 20;12:276. doi: 10.1038/s41420-026-03197-6 (PMC13283208; doi:10.1038/s41420-026-03197-6)
Supplement: Supplementary file 1 — Supplementary Materials and Methods [file 41420_2026_3197_MOESM1_ESM.docx]

Supplementary Materials and Methods

**Antibodies and chemicals**

The antibodies used in this study are listed in Supplementary Table S1. All chemical inhibitors, drugs, and the solvent (DMSO) were purchased from MedChemExpress (MCE, USA). Their respective working concentrations and treatment durations are detailed in Supplementary Table S2.

**siRNA and plasmid transfection**

The plasmid for human *RBM41* overexpression (pRP-mCherry/Neo-CAG>h*RBM41*-Flag) was purchased from VectorBuilder (Guangzhou, China). The plasmid for human *NDRG1* overexpression (pCMV-h*NDRG1*-3xFLAG-Neo) was purchased from MiaoLing (Wuhan, China). All small interfering RNAs (siRNAs), including the negative control siRNA (siCtrl), *RBM41*-targeting siRNAs (si*RBM41* #1, si*RBM41* #2), and *NDRG1*-targeting siRNAs (si*NDRG1* #1, si*NDRG1* #2), were designed and synthesized by GenePharma (Shanghai, China). The siRNA sequences are provided in Supplementary Table S3. Plasmid transfections were performed using Lipofectamine 2000 according to the manufacturer's instructions. For siRNA transfections, cells were seeded in appropriate culture dishes and transfected at 50-60% confluence using Lipofectamine 2000 at a working concentration of 1:100 (v/v, reagent to serum-free medium), with a final siRNA concentration of 50 nM. The culture medium was replaced with fresh complete medium 6-8 h post-transfection. Stable cell lines were selected and maintained using appropriate antibiotics, such as Neomycin (G418) or Puromycin, at predetermined optimal concentrations.

**CRISPR/Cas9-mediated gene knockout**

Single-guide RNAs (sgRNAs) targeting the exon encoding the RRM1 domain of *RBM41*, *NDRG1*, or the full gene were designed using the CRISPRREGN Tools (http://www.rgenome.net/cas-designer/). The oligonucleotides encoding the sgRNAs were cloned into the BbsI restriction site of the PX459 vector. The sequences of the sgRNAs used are listed in Supplementary Table S4.

**RNA isolation and** **reverse transcription quantitative PCR (RT-qPCR) analysis**

Total RNA from cells and tissues was extracted using Unizol Total RNA Extraction Reagent (Genesand Biotech, Cat: RE703) according to the manufacturer's protocol. RNA concentration and quality were determined using a NanodropTM 2000 Spectrophotometer (Thermo Fisher Scientific). cDNA was synthesized from total RNA using the UnionScript First-strand cDNA Synthesis Mix for qPCR (Genesand Biotech, Cat: SR511). Quantitative PCR (qPCR) was performed using the GS AntiQ SYBR Green Fast Mix (Genesand Biotech, Cat: SQ410). The thermal cycling conditions were as follows: 95°C for 15 mins; 40 cycles of 95°C for 15 s, 60°C for 30 s, and 70°C for 20 s. Relative gene expression levels were calculated using the 2–ΔΔCt method, with GAPDH as the internal control. Each assay was performed in triplicate. The sequences of the qPCR primers are listed in Supplementary Table S5.

**Western blot analysis**

Cells and tissues were washed with cold phosphate-buffered saline (PBS) and lysed using RIPA buffer (Meilunbio, MA0151) supplemented with protease inhibitors (Meilunbio, MB2678) and phosphatase inhibitors (Epizyme, GRF102). Lysates were incubated on ice for 30 mins and centrifuged at 12,000 rpm for 15 mins to collect the supernatant. Total protein concentration was quantified using a BCA protein assay kit (Abbkine, KTD3001). Equal amounts of protein lysates mixed with loading buffer were separated by SDS-polyacrylamide gel electrophoresis (SDS-PAGE) using Omni-Easy™ Bis-Tris gels (6−15%; PG210) and transferred onto 0.22 or 0.45 μm PVDF membranes (Merck Millipore, Cat: ISEQ00010, IPVH00010). Membranes were probed with specific primary antibodies, followed by incubation with horseradish peroxidase (HRP)-conjugated secondary antibodies. Protein bands were visualized using ECL solution (Abbkine, BMU102-CN) and imaged with an Image Quant LAS4000 chemiluminescence imaging system (GE Healthcare). Band intensity was quantified using ImageJ software (NIH, version 1.53t). All experiments were performed at least three times. Detailed antibody information is provided in Supplementary Table S1.

**RNA immunoprecipitation (RIP) assay**

The RIP assay was performed using the PureBinding® RNA Immunoprecipitation Kit (Geneseed, P0101) according to the manufacturer’s instructions with the following specific details. A total of 5 × 10⁶ SW480 cells transfected with the FLAG-RBM41 plasmid were collected and lysed in 1× Buffer A (diluted from the provided 10× Buffer A with RNase-free ddH₂O) supplemented with 1× protease inhibitor and 1× RNase inhibitor (both from the kit). For each immunoprecipitation reaction, 200 μL of Protein A+G magnetic beads were pre‑blocked with 20 μL of Buffer D (from the kit) in 1× Buffer A for 30 min at 4 °C with rotation, followed by two washes with 1× Buffer A. The beads were then incubated with 5 μg of anti‑FLAG antibody (Servicebio, GB15939‑100) or 5 μg of normal rabbit IgG (provided in the kit, 1 mg/mL) in 1 mL of 1× Buffer A for 1.5 h at 4 °C with rotation. After antibody conjugation, the beads were washed twice with 1× Buffer A. The antibody‑bead complexes were subsequently incubated with the cell lysate in 350 μL of 1× Buffer A overnight at 4 °C with rotation. Following immunoprecipitation, the beads were washed five times with 1 mL of 1× Buffer B (diluted from 10× Buffer B and supplemented with 0.01% RNase inhibitor), each wash lasting 2 min. Bound RNA was eluted with 300 μL of Buffer E (prepared by adding 1% β‑mercaptoethanol before use), treated with DNase I using DR Columns, and purified using RC Columns with Buffer F (supplemented with one volume of absolute ethanol) and Buffer G (supplemented with four volumes of absolute ethanol) according to the kit protocol. Purified RNA was then subjected to reverse transcription and qPCR analysis to determine the levels of NDRG1 and GAPDH transcripts. Primer sequences are listed in Supplementary Table S6.

**RNA pull-down assay**

DNA templates containing the T7 promoter sequence and various fragments of the NDRG1 gene were generated by PCR using human genomic DNA as the template. Primer sequences are listed in Supplementary Table S7. The PCR products were purified and used for in vitro transcription with the RNAmax‑T7 Biotin Labeling Transcription Kit (RiboBio, C11002). The transcription reaction (20 µL) contained 0.1–0.2 µg of linear DNA template, 2 µL of T7 Enzyme mix, 4 µL of 5× T7 Reaction Buffer, 6 µL of Biotin RNA Labeling Mix, and RNase‑free H₂O. The mixture was incubated at 37 °C for 3 h, followed by addition of 1 µL of DNase I (1 U/µL) and further incubation at 37 °C for 20 min to remove the DNA template. Biotinylated RNA was purified by ethanol precipitation: 10 µL of Purification Assistant A and 2 µL of Purification Assistant B were added, mixed with 300 µL of pre‑chilled absolute ethanol, and precipitated at −20 °C for 2 h. After centrifugation at 13,000 rpm for 30 min at 4 °C, the pellet was washed twice with 1 mL of 70% ethanol, air‑dried, and dissolved in 50 µL of RNase‑free water.

The pull‑down assay was performed using the PureBinding® RNA‑Protein pull‑down Kit (Geneseed, P0201). Briefly, 50 µL of Streptavidin Magnetic Beads provided in the kit were washed twice with 1× Capture Buffer (diluted from the supplied 10× Capture Buffer with DEPC‑treated water). The beads were then incubated with 50 pmol of biotinylated NDRG1 RNA probe (or a control non‑specific RNA probe) in 1× Capture Buffer at 4 °C for 30 min with rotation. After removal of the unbound probe, the RNA‑coated beads were mixed with 450 µL of cell lysate from HT29 cells. The lysate was prepared in 1× Capture Buffer supplemented with 10 µL of RNase inhibitor and 10 µL of protease inhibitor (both from the kit) and incubated on ice for 10 min, followed by centrifugation at 12,000×g for 10 min at 4 °C. The mixture was incubated overnight at 4 °C with rotation. Subsequently, the beads were washed five times with 1× Wash Buffer (diluted from 10× Wash Buffer with DEPC‑treated water and supplemented with 0.1% RNase inhibitor and 0.1% protease inhibitor), with each wash consisting of 1 mL of buffer and 1 min of vortexing. Bound proteins were eluted by adding 50 µL of 5× Loading Buffer (provided in the kit) and boiling at 100 °C for 10 min. The eluates were separated by SDS‑PAGE and analyzed by western blotting using anti‑RBM41 antibody (dilution 1:1000) and anti‑GAPDH antibody (dilution 1:5000) as a loading control.

**Annexin V/PI apoptosis assay**

Cell death was quantified using an Annexin V-FITC/PI Apoptosis Kit (MeilinBio, China). Briefly, adherent and floating cells were collected, washed with cold PBS, and resuspended in 1× Binding Buffer. The cell suspension was incubated with 10 μL of Annexin V-FITC and 5 μL of Propidium Iodide (PI) for 15 mins at room temperature in the dark. Stained cells were analyzed immediately using a BD FACS Celesta flow cytometer, and data were processed with FlowJo software (v10.8.1).

**TUNEL assay**

DNA fragmentation, indicative of cell death, was detected using the TUNEL Assay Kit (Meilunbio, China). Cells were seeded on glass coverslips in 24-well plates at a density of 2 × 10⁴ cells per well. After treatments, cells were fixed with 4% formaldehyde for 30 mins at room temperature and permeabilized with 0.3% Triton X-100 for 5 mins on ice. Coverslips were incubated with the TUNEL reaction mixture for 60 mins at 37°C in the dark, according to the manufacturer's instructions. Cell nuclei were counterstained with DAPI. Images were captured using a fluorescence microscope or laser scanning confocal microscope, and TUNEL-positive (green fluorescence) cells were counted.

**Calcein-AM/PI staining**

Cell viability was assessed using a Live and Dead Cell Double Staining Kit (Abkkine, KTA1001) according to the manufacturer's protocol. Briefly, cells were incubated with a staining solution containing 2 μM Calcein-AM and 1.5 μM PI at 37°C for 30 mins. Stained cells were observed under an inverted fluorescence microscope. Viable cells (green fluorescence) and dead cells (red fluorescence) were visualized and counted.

**RNA-seq analysis**

Total RNA was extracted from HT29 cells using Trizol reagent (Genesand, RE703) and subjected to RNA-seq analysis. All reads were uniquely mapped to genes to evaluate gene expression levels. Dysregulated genes (fold change ≥1.5, p < 0.05) were further analyzed by Gene Set Enrichment Analysis (GSEA) using the DEGseq R package.

**Staining of autophagic structures**

DALGreen and DAPRed (Dojindo Molecular Technologies, Kumamoto, Japan) were used according to the supplier's instructions. Cells transfected with specified plasmids or siRNA were pre-incubated in medium containing 1 μM DALGreen and 0.1 μM DAPRed for 30 mins. After washing off the staining medium, fluorescence images were collected using a fluorescence microscope under identical conditions.

**Transmission electron microscopy (TEM)**

For ultrastructural analysis, cells were fixed with 2.5% glutaraldehyde at 4°C for 1 h without washing the culture medium. Cells were then quickly scraped off, allowed to settle naturally into aggregates, and fixed overnight at 4°C. After washing with PBS, samples were fixed with 1% osmium tetroxide at room temperature for 2 h, followed by dehydration through a graded ethanol series and embedding in EPON812 resin. Ultrathin sections (70 nm) were prepared using a Leica UC7 ultramicrotome, stained with uranyl acetate and lead citrate, and observed under a Hitachi HT7800 transmission electron microscope operated at 80 kV.

**Immunofluorescence staining**

Indicated CRC cells were seeded on glass coverslips in 24-well plates, fixed with paraformaldehyde, permeabilized with 0.1% Triton X-100 in PBS, and blocked with 5% BSA. Coverslips were incubated with primary antibody at 4°C overnight. The following day, coverslips were incubated with fluorescently-labeled secondary antibodies for 1 h at room temperature. Cells were visualized and imaged using a fluorescence microscope.

**Hematoxylin and eosin (H&E) staining**

Paraffin-embedded tissue sections (4-5 μm) were deparaffinized and rehydrated. Sections were stained with hematoxylin for 5 mins, differentiated in 1% acid alcohol, and blued in 0.2% ammonia water. Subsequently, sections were counterstained with eosin for 2 mins, dehydrated through an ethanol series, cleared in xylene, and mounted with neutral balsam. Histopathological evaluation was performed by two experienced pathologists using a microscope.

**Immunohistochemistry (IHC)**

Tissue samples were fixed with formalin and embedded in paraffin. Tissue sections were deparaffinized, rehydrated, and subjected to antigen retrieval. Endogenous peroxidase activity was blocked by incubation with 3% hydrogen peroxide. After blocking with 10% normal goat serum for 1 h at room temperature, sections were incubated with primary antibodies at 4°C overnight, followed by incubation with HRP-conjugated secondary antibody for 30 mins at room temperature. Immunoreactivity was detected using 3,3'-diaminobenzidine (DAB), and sections were counterstained with hematoxylin. Images were captured under a microscope.

**Cell proliferation and colony formation assays**

The effect of *RBM41* overexpression or knockdown/knockout on cell proliferation was measured by CCK-8 assay. Cells were seeded into 96-well plates in triplicate at a density of 3000 cells per well. Cell viability was measured at indicated time points using the Cell Counting Kit-8 (MCE, HY-K0301) according to the manufacturer's instructions.

The effect of *RBM41* on colony-forming ability was determined by colony formation assay. Cells were seeded into 6-well plates at a density of 200 cells per well and cultured for 14 days. Colonies were fixed with paraformaldehyde and stained with crystal violet for 10 mins. Each experiment was repeated at least three times.

**Nuclear/cytoplasmic protein extraction**

Nuclear and cytoplasmic proteins were extracted using a Nuclear and Cytoplasmic Protein Extraction Kit (Beyotime, Cat: P0027) according to the manufacturer's instructions. Briefly, transfected cells were washed with cold PBS, centrifuged at 800 rpm for 5 mins, and resuspended in Cytoplasmic Protein Extraction Reagent A. After incubation on ice for 15 mins, Cytoplasmic Protein Extraction Reagent B was added, and samples were centrifuged at 12,000 × g for 15 mins. The supernatant contained the cytoplasmic fraction. The pellet was resuspended in Nuclear Protein Extraction Reagent, incubated on ice for 30 mins, and centrifuged at 12,000 × g for 15 mins. The supernatant contained the nuclear fraction. Protein concentration was determined using a BCA Kit (Abbkine, Cat: KTD3001).

**Xenograft tumor experiments**

Five-week-old female BALB/c nude mice were obtained from Liaoning Changsheng Biotechnology Co., Ltd. and maintained under specific-pathogen-free (SPF) conditions. Animals were randomly assigned to experimental groups using a lottery method and subcutaneously inoculated with 100 μL of PBS containing 5.0 × 10⁶ cells. Beginning 7 days post-inoculation, tumor dimensions (longest and shortest diameters) were measured every 2 days. Mice were euthanized on day 27, and tumor weights were recorded. Mice were monitored daily for signs of distress, and humane endpoints were strictly applied in accordance with the Guidelines for the Care and Use of Laboratory Animals in Cancer Research. If mice failed to develop palpable tumors (longest diameter <2 mm) by 14 days post‑inoculation, or exhibited severe illness (body weight loss >20%, inability to eat or move spontaneously) or tumor ulceration/infection, they were excluded. Excised tumor tissues were fixed in 4% paraformaldehyde, processed for dehydration, paraffin-embedded, and sectioned. Paraffin-embedded sections were subjected to H&E staining for histopathological evaluation and to Ki-67 immunohistochemistry to assess tumor cell proliferation.

**PDO models culture**

Fresh human colorectal cancer tissues were collected from surgical resection, placed at 4 °C, and transported to the laboratory within 12 h. Tissues were washed three times with ice‑cold DPBS containing 1% penicillin‑streptomycin and 0.5 mg/mL amphotericin B, then minced into 1–3 mm³ fragments. The fragments were digested in DMEM/F12 containing 1 mg/mL collagenase/dispase, 1% penicillin‑streptomycin, and 0.5 mg/mL amphotericin B at 37 °C for 15 min with shaking at 110 rpm. Digestion was stopped by adding three volumes of ice‑cold Primary Culture Buffer (absin, abs9445), then triturated, filtered through a 100 µm strainer, and centrifuged at 300×g for 5 min at 4 °C. The pellet was mixed with ice‑cold Matrigel (absin, abs9495) at a ratio of 25 µL Matrigel per 1 µL pellet. Aliquots of 25 µL were dispensed into a pre‑warmed 24‑well plate and solidified by inverting at 37 °C for 30–40 min. After gelation, 600 µL of human colorectal cancer organoid medium (absin, abs9523) was added per well. Medium was refreshed every 2–3 days. Organoids were passaged every 10–14 days at a 1:2 split ratio using Organoid Passage Digestion Solution (absin, abs9445) for 2–3 min at 37 °C, followed by termination with five volumes of ice‑cold Passage Buffer (absin, abs9445).

**Drug treatment and IC_50_ assessment in PDOs**

After organoid establishment, organoids were harvested, dissociated, and seeded into 96‑well plates at 5 µL of Matrigel per well. After solidification, 100 µL of organoid medium was added to each well. Three days later, the organoids were treated with serial dilutions (0, 0.01, 0.1, 1, 10, 100 µM) of 5‑fluorouracil, oxaliplatin, or irinotecan for 7 days. Each concentration was tested in triplicate. Following treatment, cell viability was assessed using the ATP Assay Kit (Meilunbio, MA0440). Briefly, cells were lysed with the provided ATP assay buffer, and the lysate was directly transferred to a white‑walled, clear‑bottom 96‑well plate. The ATP reaction mix was added, and luminescence was measured immediately using a microplate reader. The half‑maximal inhibitory concentration (IC_50_) was calculated using GraphPad Prism software.

**Nascent RNA detection**

Indicated cells were treated with 500 μM 5-ethynyluridine (EU) (Ribobio, C10316) for 2 h to label newly synthesized RNA. For nascent RNA capture, EU-labeled RNAs were biotinylated with 0.25 M biotin azide (Ribobio, C00101) in Click-iT reaction buffer. Biotin-conjugated, EU-labeled RNA was pulled down, and nascent *NDRG1* RNA levels were measured by RT-qPCR.

**RNA decay assay**

Indicated cells were seeded into 12-well plates and treated with actinomycin D (5 μg/mL) for 0, 3, 6, 9, and 12 h. Cells were lysed with Trizol reagent for RNA extraction. RT-qPCR analysis was performed. The mRNA half-life was calculated based on RNA concentration and degradation rate.

**Statistical analysis**

All quantitative data are presented as the mean ± standard deviation (SD) from at least three independent biological replicates, unless otherwise specified in the Figure legends. Statistical analyses were performed using GraphPad Prism software (version 8.0.0). The specific statistical tests used for each experiment are detailed in the corresponding Figure legends. Briefly, comparisons between two groups were analyzed using the unpaired, two-tailed Student's t-test. For data involving two independent variables, two-way ANOVA with Sidak's multiple comparisons test was used. P values are indicated in the Figures.
