# Supplementary material for: RNA binding motif protein RBM41 promotes colorectal tumorigenesis by impeding the maturation of NDRG1 pre-mRNA
Source: Cell Death Discov. 2026 Jun 20;12:276. doi: 10.1038/s41420-026-03197-6 (PMC13283208; doi:10.1038/s41420-026-03197-6)
Supplement: Supplementary file 2 — Full and Uncropped Western Blots [file 41420_2026_3197_MOESM2_ESM.docx]

**
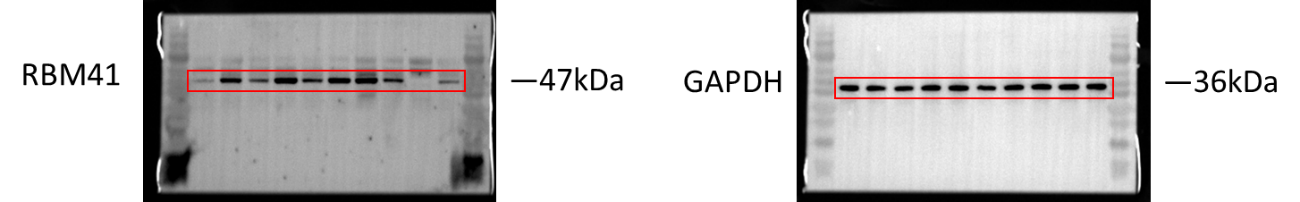
**

**Uncropped images corresponding to those shown in Figure 1H**

**
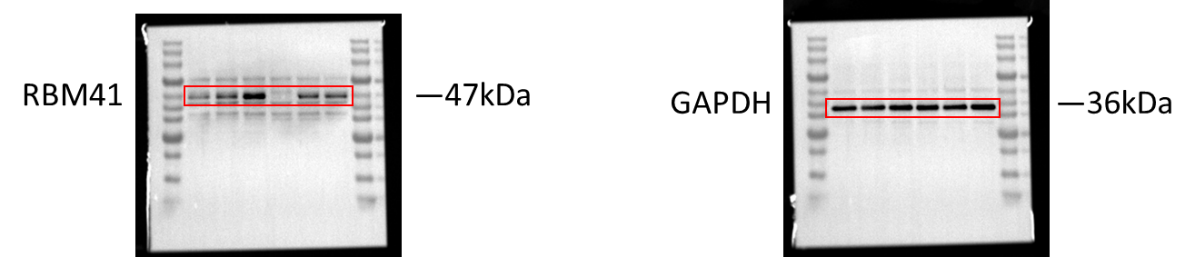
**

**Uncropped images corresponding to those shown in Figure 2B**

**
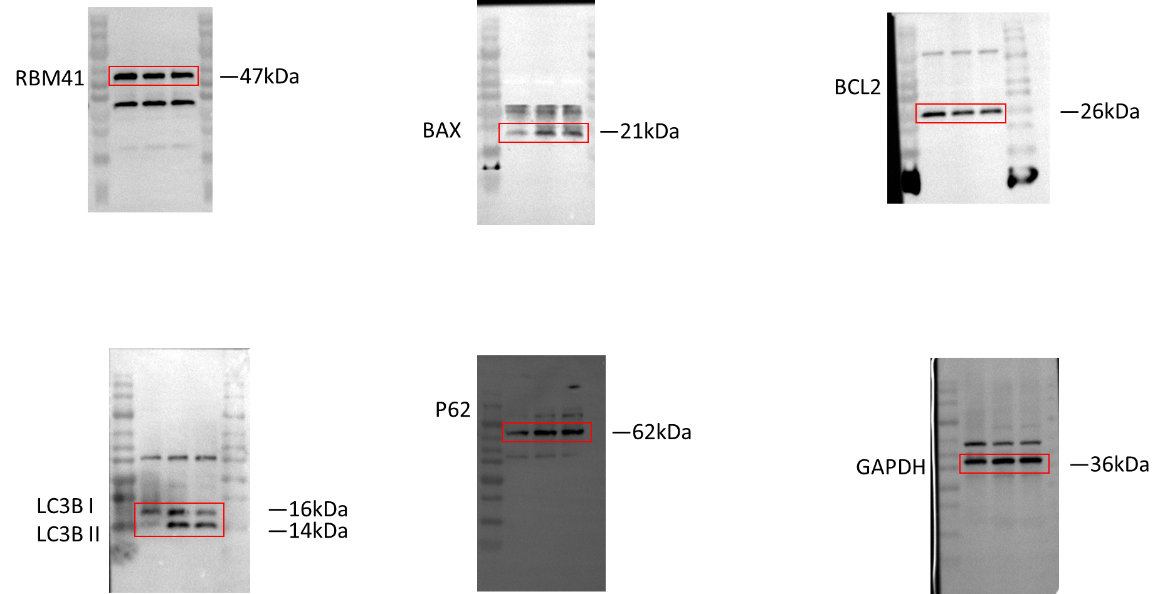
**

**Uncropped images corresponding to those shown in Figure 3B**

**
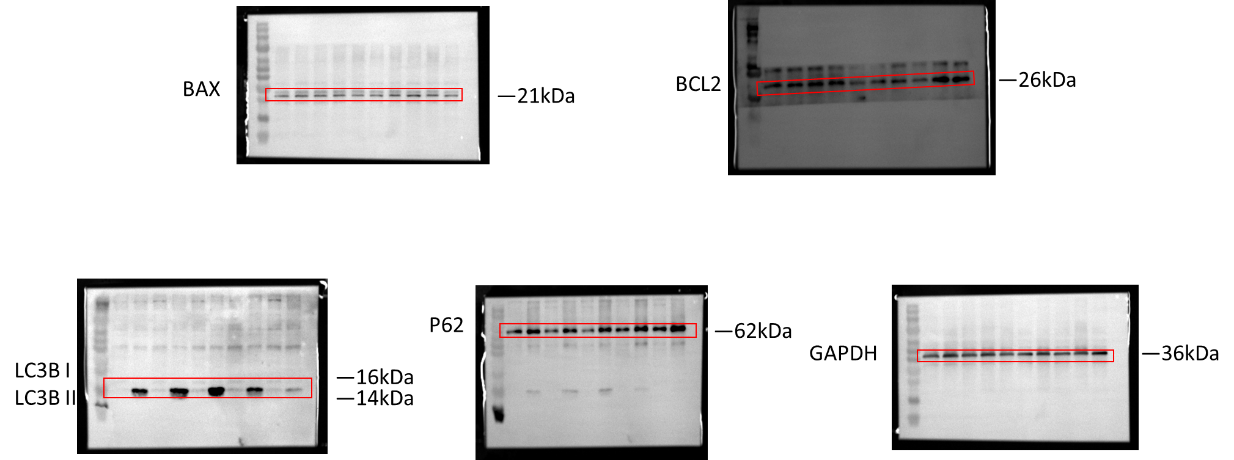
**

**Uncropped images corresponding to those shown in Figure 3G**

**
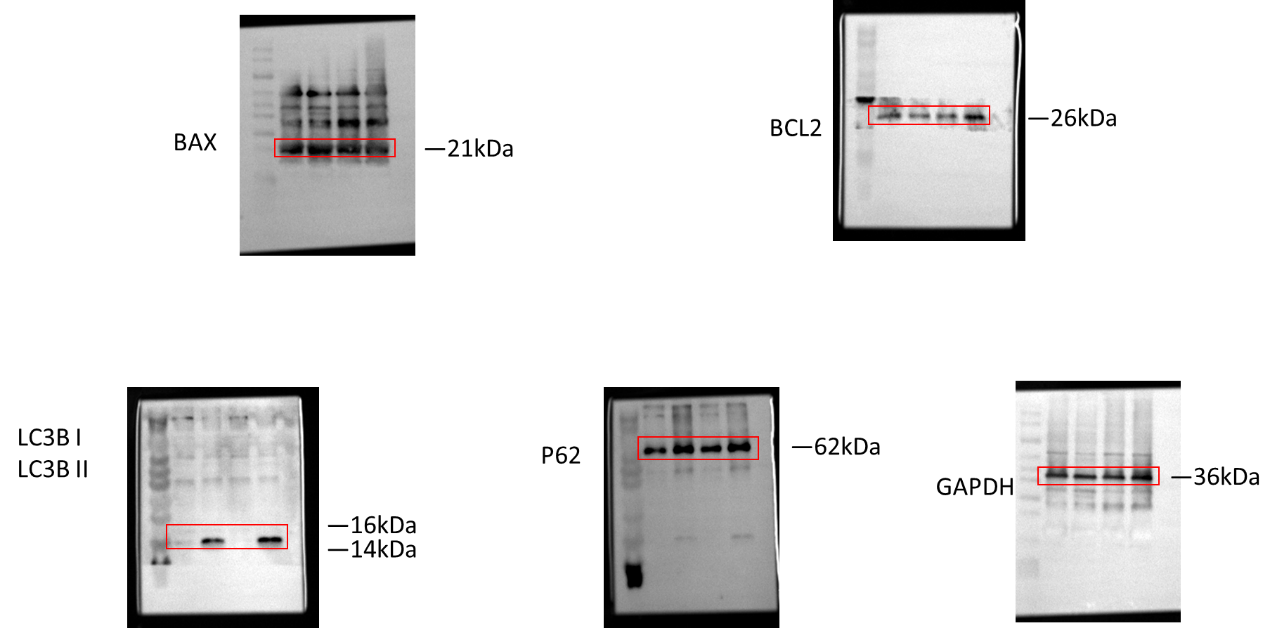
**

**Uncropped images corresponding to those shown in Figure 3H**

**
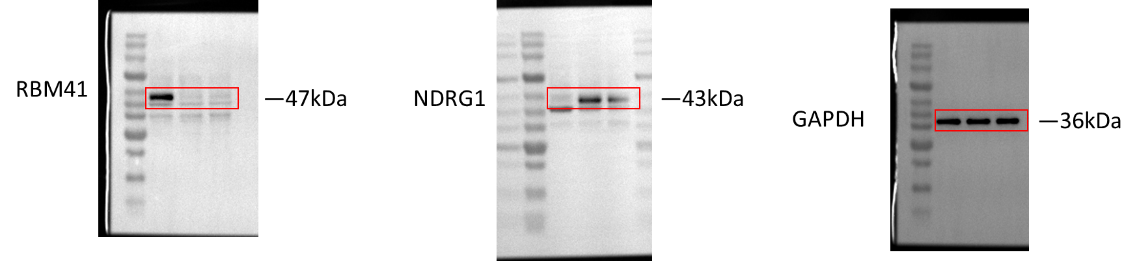
**

**Uncropped images corresponding to those shown in Figure 4C(left)**

**
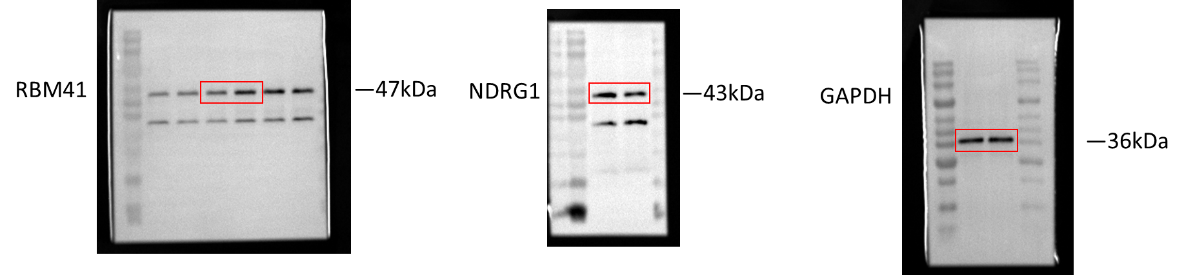
**

**Uncropped images corresponding to those shown in Figure 4C(right)**

**
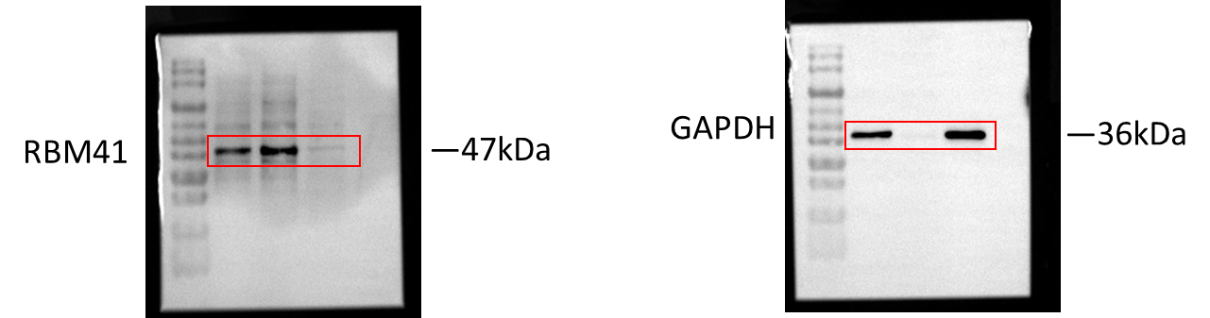
**

**Uncropped images corresponding to those shown in Figure 4F**

**
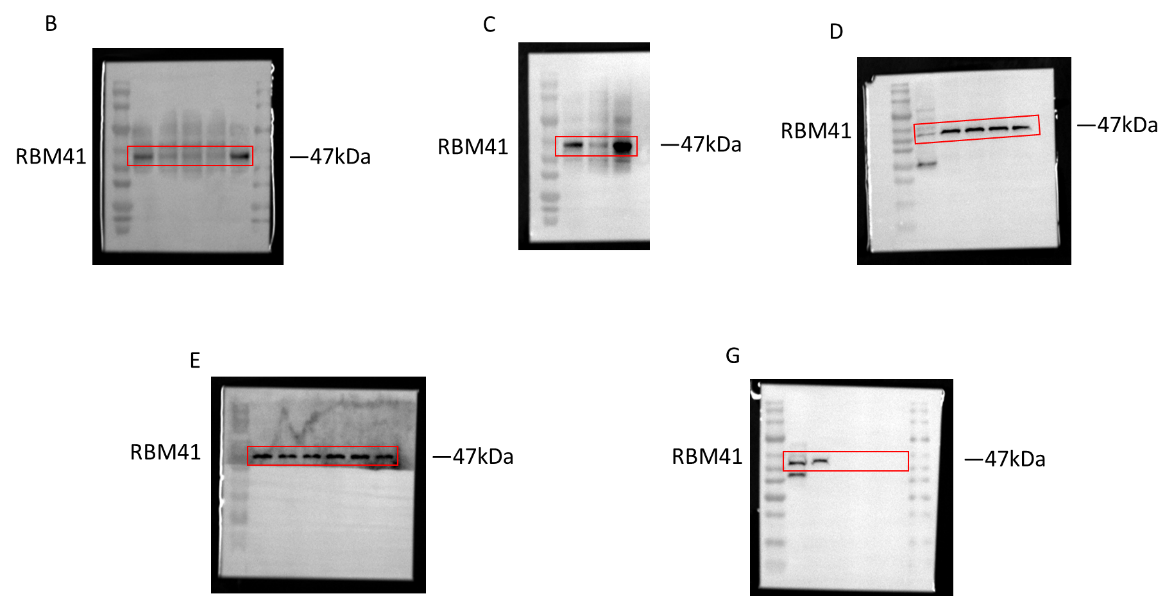
**

**Uncropped images corresponding to those shown in Figure 5**

**
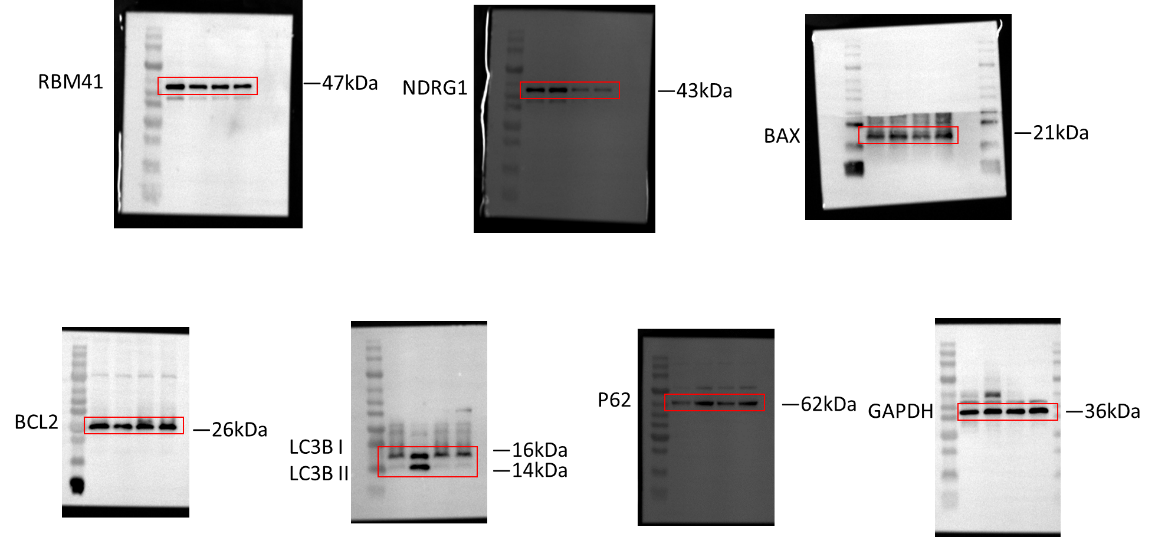
**

**Uncropped images corresponding to those shown in Figure 6B**

**
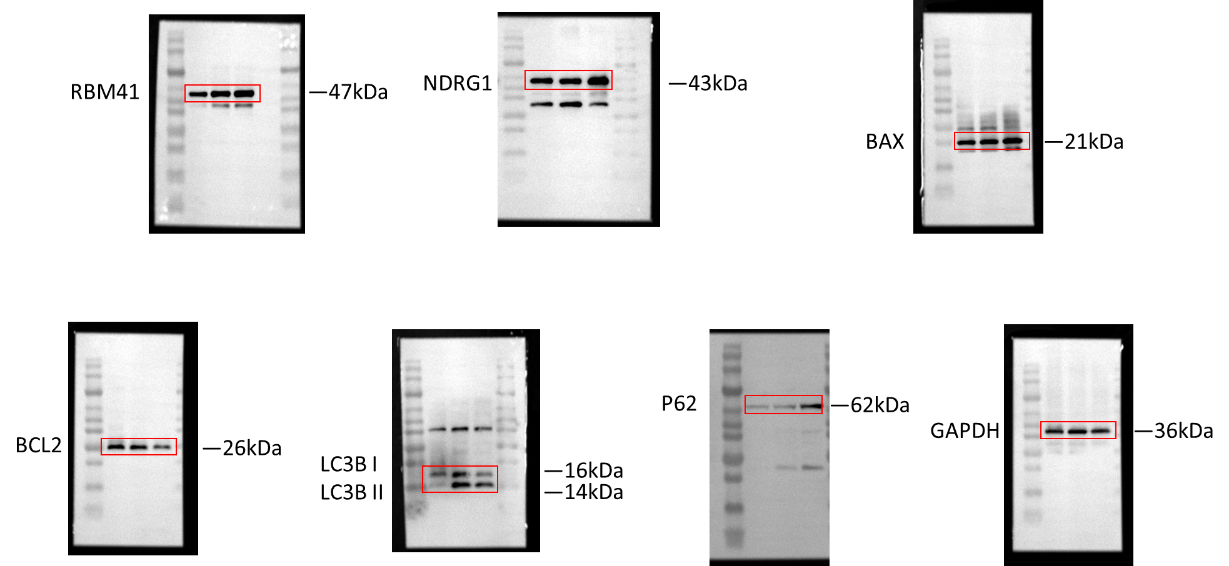
**

**Uncropped images corresponding to those shown in Figure 6H**
